# Supplementary material for: Model-free pattern separation of two-color ultrafast X-ray diffraction
Source: Nat Commun. 2026 Sep 3;17:10465. doi: 10.1038/s41467-026-77085-z (PMC13635185; doi:10.1038/s41467-026-77085-z)
Supplement: Supplementary file 1 [file 41467_2026_77085_MOESM1_ESM.pdf]

Supplementary Information for: Model-free pattern separation of two-color ultrafast X-ray diffraction

Linos Hecht*,[1] Yevheniy Ovcharenko*,[2] Asbjørn Ø. Lægdsmand,[3] Björn Bastian,[4] Thomas M. Baumann,[2] Alessandro Colombo,[1] Subhendu De,[5] Alberto De Fanis,[2] Simon Dold,[2] Thomas Fennel,[6] Robert Hartmann,[7] Katharina Kolatzki,[1] Sivarama Krishnan,[5] Björn Kruse,[6] Aaron C. Laforge,[8] Bruno Langbehn,[9] Suddhasattwa Mandal,[10] Tommaso Mazza,[2] Cristian Medina,[11] Christian Peltz,[6] Thomas Pfeifer,[11] Björn Senfftleben,[1,2] Keshav Sishodia,[5] Frank Stienkemeier,[12] Rico Mayro P. Tanyag,[13] Paul Tümmler,[6] Sergey Usenko,[2] Andreas Heidenreich,[14,15] Michael Meyer,[2] Daniela Rupp*,[1] and Marcel Mudrich*[3,16]

[1]*Laboratory for Solid State Physics, ETH Zurich, 8093 Zurich, Switzerland**
[2]*European XFEL, Holzkoppel 4, 22869 Schenefeld, Germany*
[3]*Institute of Physics, University of Kassel, 34132 Kassel, Germany*
[4]*Wilhelm Ostwald Institute for Physical and Theoretical Chemistry, Leipzig University, 04103 Leipzig, Germany*
[5]*Quantum Center of Excellence for Diamond and Emergent Materials and Department of Physics, Indian Institute of Technology Madras, Chennai 600036, India*
[6]*Institute of Physics, University of Rostock, 18059) Rostock, Germany*
[7]*PNSensor GmbH, 81739 Munich, Germany*
[8]*Physics Department, University of Connecticut, Storrs, Connecticut 06269-3046, United States*
[9]*Institute for Optics and Atomic Physics, Technical University Berlin, 10623 Berlin, Germany*
[10]*Indian Institute of Science Education and Research Pune, Pune 411008, India*
[11]*Max Planck Institute for Nuclear Physics, 69117 Heidelberg, Germany*
[12]*Institute of Physics, University of Freiburg, 79104 Freiburg, Germany*
[13]*Department of Chemistry, Aarhus University, 8000 Aarhus C, Denmark*
[14]*Kimika Fakultatea, Euskal Herriko Unibertsitatea (UPV/EHU) and Donostia International Physics Center (DIPC), P.K. 1072, 20080 Donostia, Spain*
[15]*IKERBASQUE, Basque Foundation for Science, 48011 Bilbao, Spain*
[16]*Center for Interdisciplinary Nanostructure Science and Technology (CINSaT), University of Kassel, 34132 Kassel, Germany*

Detector corrections

We thoroughly processed the diffraction patterns before applying the pixel-based separation method. How the detector works is described in detail in [38]. Below, the correction steps are listed and briefly described. The first two steps are applied to all data; all further steps are updated during the experiment:

1. **Charge transfer efficiency** map: As the charge is read out at one of the two lateral edges, every pixel's charge needs to be transported to the outside. In doing this, some charge is lost during the transport process. Using the known charge transfer efficiency, the signal of the more central columns is increased accordingly.
2. **Gain** map: Every detector row has an individual gain. Before the experiment, we created a homogeneous signal through fluorescence at a signal level where, on average, only single photons are sparsely distributed on the detector. Collecting row-wise all events with only a single pixel activated creates an ADU distribution for every row. The gain map multiplies every row so that the average ADU value of a single photon is the same for all rows.
3. **Background** pattern: Regularly, we took background runs, meaning no scattering target was introduced into the interaction region. We take 20000 patterns and delete every pixel's brightest and darkest ten values. Afterward, the remaining values are averaged. This background pattern is then subtracted from the data.
4. **Mask**: As the detector already has some damaged areas, we mask some parts. We use the mean and standard deviation of every pixel to determine which pixels to mask. Generally, we mask generously by choosing strict values and excluding surrounding pixels, as we want to support pixel-based separation as much as possible. We exclude a pixel if
   (a) the mean value is above a threshold (hot pixel).
   (b) the standard deviation is too big (flickering pixel) or too small (stuck pixel).
   (c) the ratio between mean and standard deviation is too big or too small.
5. **Common-mode correction**. During the readout process of a single pattern, the electronics are subjected to noise. This results in a different baseline for every column of pixels. As this is constant for the entire column, it can be removed afterward by subtracting the median of each column, provided the signal does not cover most of the column.
6. **Noise rejection**: We create a noise map from the background run. After background subtraction and common-mode correction of each pattern, we calculate

* lhecht@phys.ethz.ch, yevheniy.ovcharenko@xfel.eu, ruppda@ethz.ch, mudrich@uni-kassel.de

the standard deviation of every pixel. If a pixel's value in the data is below twice the standard deviation, we mark it as detector noise and remove it.

7. **Detector plate positioning**. The detector halves are movable in the detector plane and were moved to reduce stray light on the detector. Using spherical hits as a reference, we add pixels in the center of the pattern matrix and align the plates laterally by adding blank pixels at the edges.

8. **Center finding**. The exact center of the scattering pattern fluctuates from shot to shot. It depends on the position of the HND in the pulse's bent wavefront. We fit a center position to every pattern using the point symmetry of the scattering pattern.

Pixel-based cluster assignment

The processed raw patterns are passed through a series of steps to classify the pulse components of different parts of the pattern. First, the clusters of bright pixels in each pattern are labeled, noting the total brightness and their position. The relative intensity of the red and blue pulses is estimated. Finally, each cluster is assigned photon components based on its ADU value.

Split events, where photons partly activate more than one neighboring pixel, comprise about a third of all single photon hits in flat illumination. The image detection of the clusters was done in two steps. The one-photon signal can only create specific 1-4 pixel clusters. These were first labeled by convolving the pattern with matrices chosen to capture each shape. Afterwards, the remaining clusters were identified using a standard connected component analysis algorithm in SciPy.

Because each diffraction pattern can result from scattering off droplets anywhere between the two foci of the pulses, the resulting patterns contain different ratios of red and blue photons. Using the histogram of the cluster ADU values, similar to Fig. 5, the relative sizes of the one-photon and noise peaks are found. For these fits on individual patterns, the heights and the positions of the peaks are varied. The other parameters determining their shape were assumed to be the same for all patterns.

We now consider the signal in each cluster as resulting from a single-photon or multi-photon event, with a mean value of a photon μ and a standard deviation σ. Under the assumption that the signal from each pump or probe photon is normally distributed (N), the resulting distribution $S_{\mathrm{n,\ m}}$ of a given multiphoton event can be estimated.

$$\begin{aligned} S_{\mathrm{pump}} &\sim N(\mu_{\mathrm{pump}}, \sigma^2_{\mathrm{pump}}) \\ S_{\mathrm{probe}} &\sim N(\mu_{\mathrm{probe}}, \sigma^2_{\mathrm{probe}}) \\ S_{n,m} &\sim N(n\mu_{\mathrm{pump}} + m\mu_{\mathrm{probe}}, n\sigma^2_{\mathrm{pump}} + m\sigma^2_{\mathrm{probe}}) \\ &\quad \text{for } n, m \in \mathbb{N}. \end{aligned}$$

For each cluster with its associated ADU value x_i, we calculate the $S_{n,m}(x_i)$ using the pattern-specific one-peak heights and widths found by fitting Gaussians to the ADU histogram. The highest of these $S_{n,m}(x_i)$ is assumed to be the origin of the cluster. Summing all these different $S_{n,m}(x_i)$ gives a measure of the total value attributed to this cluster, from all considered combinations. A certainty value for the found combination is estimated by dividing the highest $S_{n,m}(x_i)$ by the total. The reconstructed components of all clusters with a certainty above a set threshold are then used to construct a red-only and a blue-only pattern.

Estimation of the probability of multiphoton events

In the case of a recorded ADU value compatible with a signal of more than a photon, proper probability coefficients must be assigned to maximize the likelihood for a correct identification of the color's contribution. In this section, we discuss the case of two-photon events for the sake of simplicity, and the conclusions drawn here can be easily extended to higher photon counts.

When a single photon is recorded, the probability p of such a photon to be red is determined by the relative intensity of the red scattered field at the specific coordinates i, j of the diffraction pattern, i.e.:

$$p_{ij} = \frac{I^R_{ij}}{I^R_{ij} + I^B_{ij}} \tag{1}$$

where I^R and I^B are the intensities of the red and blue fields, respectively. The probability of recording a blue photon is, thus, $1 - p_{ij}$.

The value of p_{ij} fully determines the probabilities of multiple photon events. In particular, for a two-photon event, three probabilities have to be calculated: p^{RR}_{ij} (two red photons), p^{BB}_{ij} (two blue photons), and p^M_{ij} (a mixed event). Their value is determined by a binomial distribution for two trials of independent events, that is:

$$\begin{aligned} p^{RR}_{ij} &= p^{RR}(p_{ij}) = p^2_{ij} \\ p^{BB}_{ij} &= p^{BB}(p_{ij}) = (1 - p_{ij})^2 \\ p^M_{ij} &= p^M(p_{ij}) = 2\, p_{ij}\, (1 - p_{ij}) \end{aligned} \tag{2}$$

The probability of the two-photon events depends on the local probability p_{ij}, which requires the knowledge of the two intensity contributions $I^R_{ij} + I^B_{ij}$, as shown in Eq. (1). However, this information is inaccessible, and its retrieval is the primary focus of the separation method presented in this work. As a consequence, the coefficients in Eq. 2 have to be assigned *a priori* and have to be agnostic of the local intensity of the fields.

The most straightforward option is to replace local probabilities with the average value:

$$\hat{p}^X = \frac{1}{N^2} \sum_{i,j=1}^{N} p^X_{ij} \sim \int_0^1 p^X(p)\, w(p)\, dp \tag{3}$$

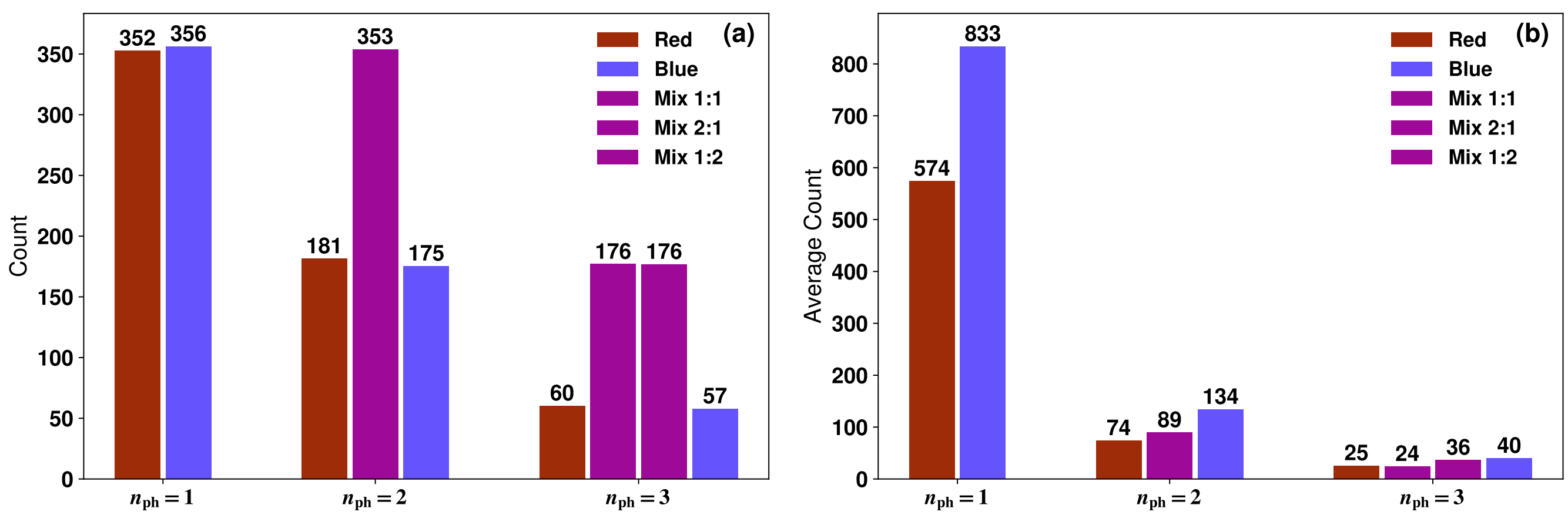

Supplementary Fig. 1. Histogram of photon counts per pixel on simulated data using the experimental photon energies. The number of photons in a pixel with all possible color combinations is shown as bars (see legend). (a) a flat field with an average photon density of 1. It reproduces the binomial distribution. (b) is an averaged result for diffraction patterns of varying radius (200 to 350 nm in steps of 50 nm) with the same total scattered intensity. Due to the continuously varying local intensity ratio across the scattering angle, a different photon distribution is observed.

where N is the linear dimension of the diffraction pattern. The function $w(p)$ is a *weight* function that expresses the relative abundance of pixels with a specific red probability p, i.e., a specific relative intensity of the red signal (through Eq. (1)).

The integral form on the right-hand side of Eq. (3) allows the approximation of $\hat{p}^{RR}$, $\hat{p}^{BB}$ and $\hat{p}^{M}$ through assumptions on "how often" the specific probability p occurs in the diffraction data, i.e., on the weighting function $w(p)$.

For example, in the case of *flat fields* (i.e., constant field intensity throughout the whole diffraction signal), only a single probability value p_0 is expected at all coordinates ij, $w(p) = \delta(p - p_0)$. As expected, when inserted into Eq. (3), it directly provides the canonical binomial coefficients $\hat{p}^{RR} = p_0^2$, $\hat{p}^{BB} = (1 - p_0)^2$, and $\hat{p}^{M} = 2\,p_0\,(1 - p_0)$. Specifically, for equal intensities of the two fields, the mixed photon event $\hat{p}^{M}$ is twice as probable as the single-color two-photon events $\hat{p}^{RR}$ and $\hat{p}^{BB}$. The simulation of such a homogeneous intensity distribution with a flat-field indeed yields the expected behavior, as illustrated in Supplementary Fig. 1a. Here, the simulation is performed with equal intensities between the two colors. For the two-photon case, the mixed events have a count rate twice that of the single-color two-photon events.

However, the *flat-field* assumption is significantly different from the behavior of the intensities expected in a diffraction pattern. Due to the isolated nature of the samples, the diffraction intensities oscillate as a function of momentum transfer $\vec{q}$ between 0 (known as *nodes* of the scattered field) and a maximum value that decreases with increasing momentum transfer ($\propto |\vec{q}|^{-4}$ for hard spheres). Furthermore, the oscillation frequency of the signal on the detector is different between the two colors, due to the different wavelengths. For this reason, independent of the overall intensity ratio between the two colors, the *weight* function $w(p)$ is non-zero in the whole interval $p \in [0, 1]$. Specifically, there are always coordinates at which $I_{ij}^{B} \sim 0$ and $I_{ij}^{R} > 0$, corresponding to $p_{ij} = 1$ as well as the inverse case, where $p_{ij} = 0$. Due to the continuity of the scattering intensity, all other p values are expected throughout the diffraction pattern.

In the case of similar overall intensities of the two colors, a model for $w(p)$ more accurate than the *flat-field* discussed before can be obtained by assigning equal weights to all p values, i.e., $w(p) = 1$. In this case, the expectation values $\hat{p}^{RR}$, $\hat{p}^{BB}$, and $\hat{p}^{M}$ can be calculated through Eq. (3) as:

$$\hat{p}^{RR} = \hat{p}^{BB} = \hat{p}^{M} = \frac{1}{3} \tag{4}$$

This conclusion can be further extended for cases where the overall intensity contribution of the two colors is not equal, i.e., $\sum_{ij} I_{ij}^{B} = \alpha \sum_{ij} I_{ij}^{R}$ for $\alpha \neq 1$. In these cases, the strictly non-zero nature of $w(p)$ throughout the interval $[0, 1]$ is retained, with 0 and 1 still occurring at $I_{ij}^{R} = 0$ and $I_{ij}^{B} = 0$, respectively, independently of the scaling factor α. Still, $\alpha < 1$ increases the chances of $I_{ij}^{R} > I_{ij}^{B}$ locally (as the red signal is brighter), thus increasing $w(p)$ in the interval $[0, 0.5]$ and decreasing $w(p)$ in $[0.5, 1]$. The opposite behavior is expected in the case of $\alpha > 1$. In those cases, the following relation approximately holds:

$$\min[\hat{p}^{RR}, \hat{p}^{BB}] \lesssim \hat{p}^{M} \lesssim \max[\hat{p}^{RR}, \hat{p}^{BB}] \tag{5}$$

These conclusions are reached with very generic assumptions about the shape of the scattered field, and in particular just the presence of zeros in the intensity contributions of the two colors and their similar decay as a function of the transfer momentum $|\vec{q}|$. The suitability of the condition deduced in Eq. (5) is directly verified in Supplementary Fig. 1b, which reports the histogram of the photon distributions up to three photon events for a simulated two-color diffraction pattern of a helium droplet with uneven color contributions.

The addition of more assumptions to achieve more restrictive conditions in $\hat{p}^{RR}$, $\hat{p}^{BB}$, and $\hat{p}^{M}$ would impact the generality of the pixel-based separation. For this reason, $\hat{p}^{RR}$ and $\hat{p}^{BB}$ are inferred directly from the ratio of single-photon events. The coefficient assigned to the mixed-color event is directly estimated as the average of $\hat{p}^{RR}$ and $\hat{p}^{BB}$, to fulfill the condition in Eq. (5) without lacking generality. The same conclusions drawn here for two-photon events can be extended to higher photon counts, though these are less relevant to the overall performance of the separation algorithm.

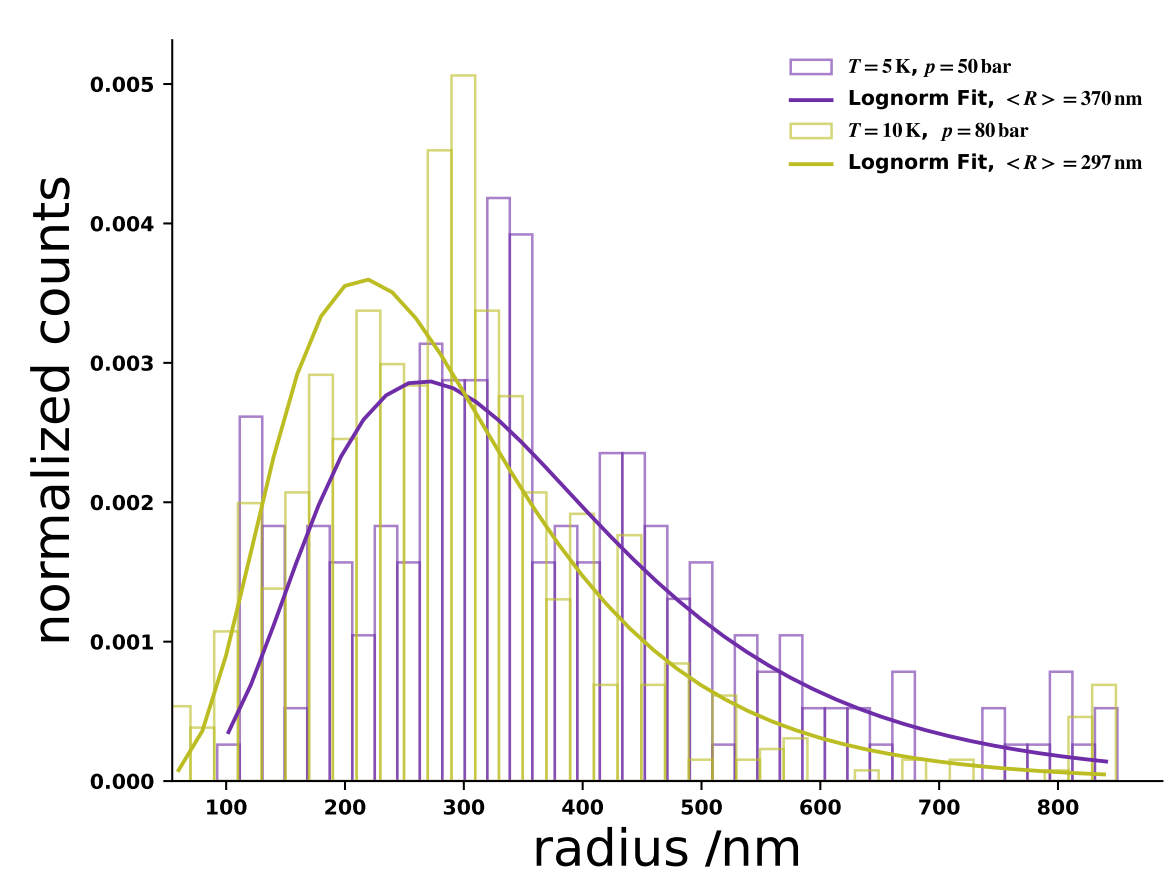

Supplementary Fig. 2. Size distribution of HNDs produced at two different valve temperatures T and expansion pressures p, see the legend. For a pulsed nozzle, a lognormal distribution is assumed.

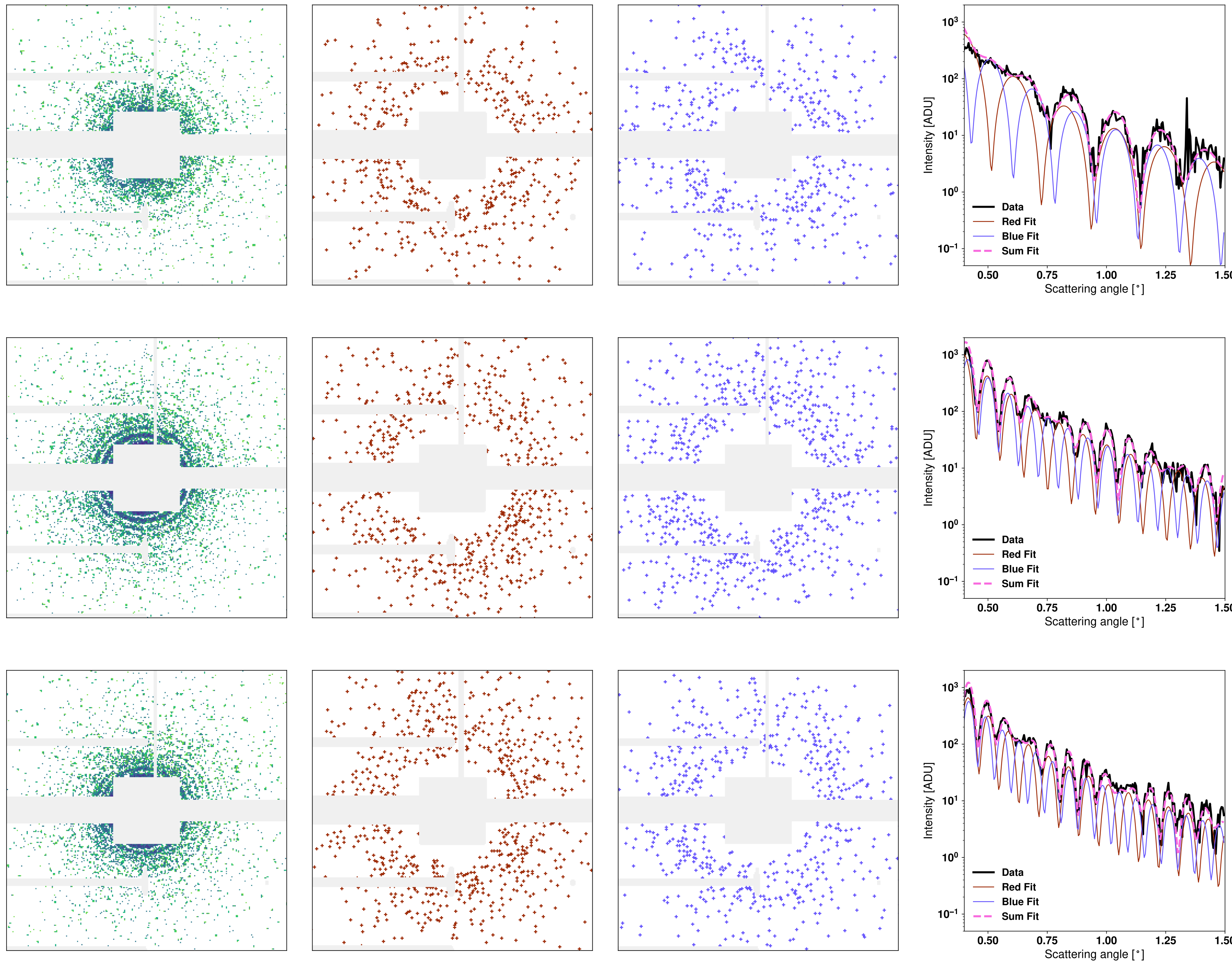

Supplementary Fig. 3. Sample diffraction patterns and radial intensity profiles including Mie fits. Colormaps correspond to Figs. 1 and 2. The first column shows the full diffraction pattern. The second and third columns show the color-separated patterns. Here, we chose a low certainty threshold (1%) to show the full separation capability. The last column depicts the radial intensity profiles; black lines are the original data, the pink dashed lines are the Mie fits, and the blue and red solid lines are the two color components resulting from the fit. Rows are ordered by HND size: $R = 173,\ 354,\ 423\,\mathrm{nm}$.

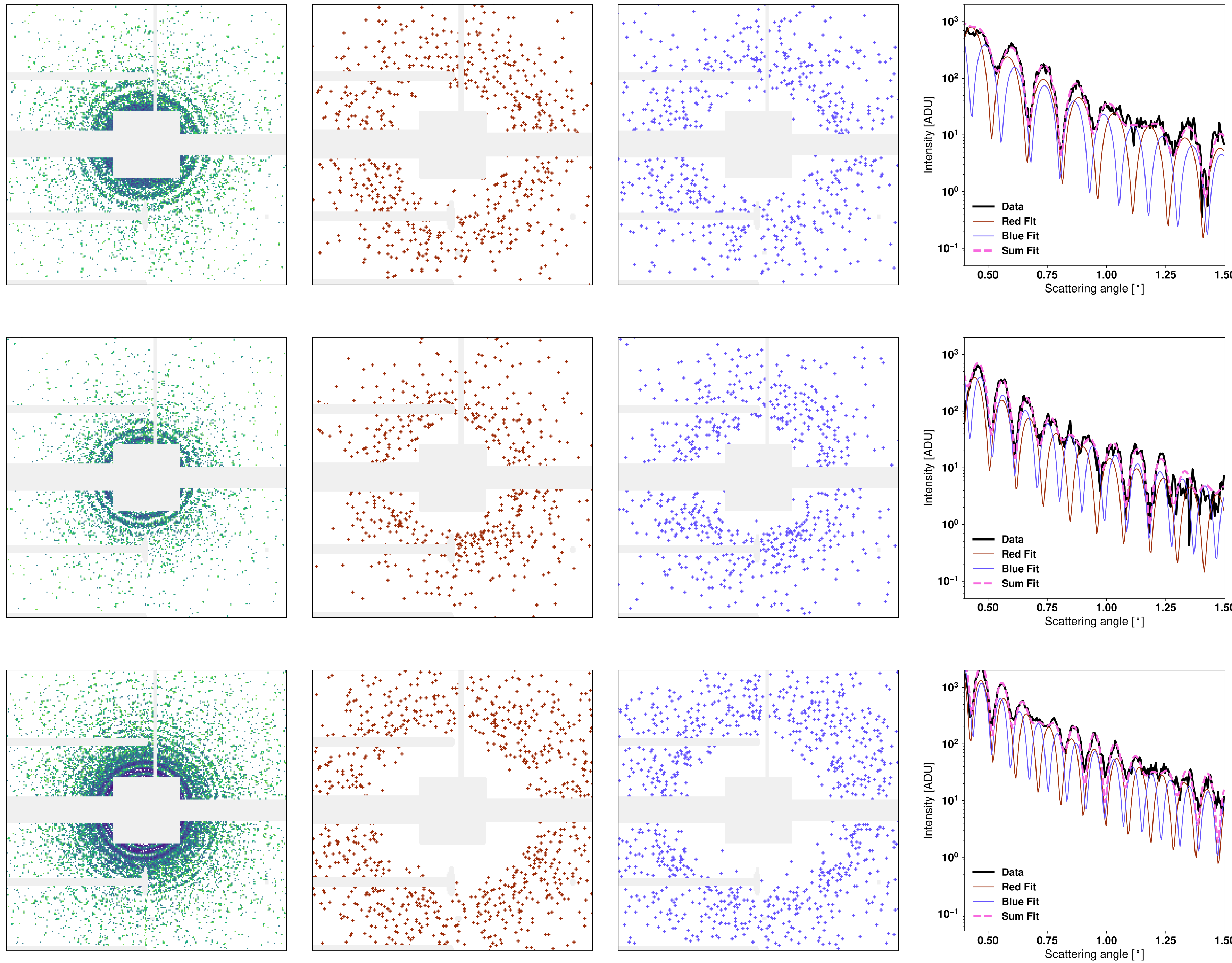

Supplementary Fig. 4. Sample diffraction patterns and radial intensity profiles including Mie fits: Figure is structured as Supplementary Fig. 3. Rows are ordered by HND size: $R = 239,\ 314,\ 374\,\mathrm{nm}$.

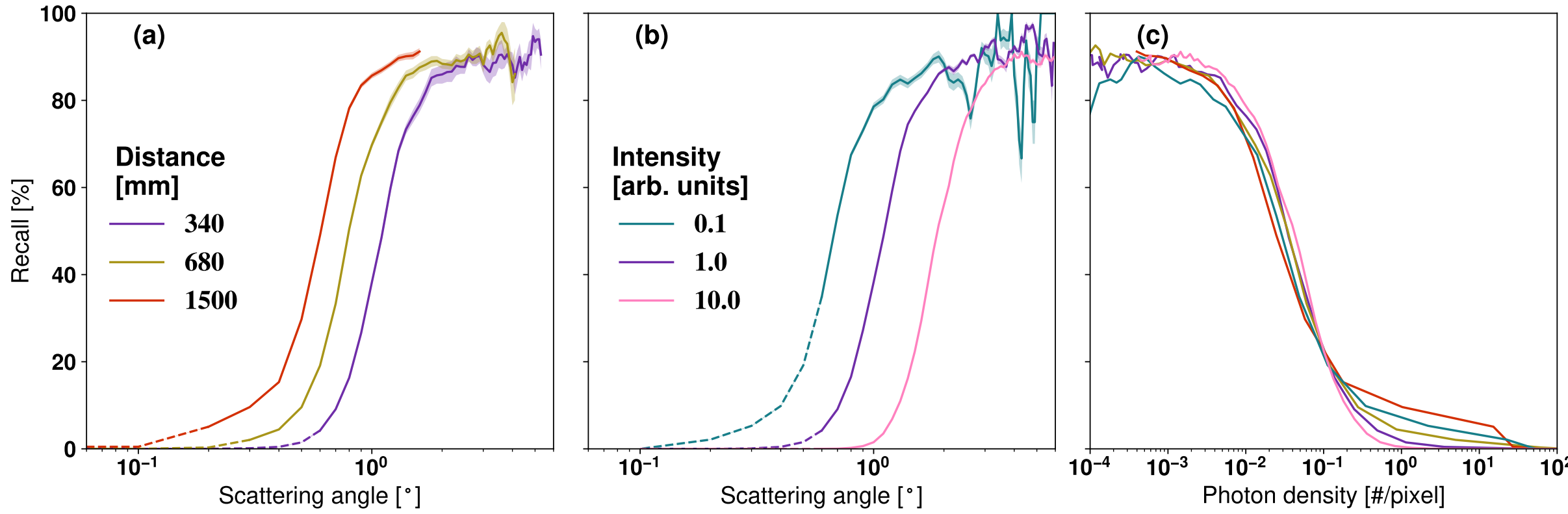

Supplementary Fig. 5. (a) Recall (efficiency of correctly assigning pixel values to noise or to single or multiple red and blue photons) as a function of the scattering angle. Three different positions of the detector with respect to the interaction point are assumed. The experiment's distance was 340 mm. (b) Recall for various intensities of the pulses relative to the experimental value set to 1. Dashed lines indicate the ranges of angles that are excluded by the gap between detector modules in the experiment. (c) Same data as in (a) and (b) replotted as a function of the area density of photons on the detector.

Influences on the recall

The recall is not constant throughout the entire diffraction pattern but rather depends on the local signal intensity. Supplementary Fig. 5 shows the recall as a function of the scattering angle for different values of the distance between the detector and the interaction point (a) and for different values of the intensity of the pulses relative to the experimental value (b). The experimental conditions are specified with a distance of 340 mm and an intensity value of 1.

The area of high recall could in principle be extended by increasing the distance to the detector, Supplementary Fig. 5a, and by reducing the intensity of scattered light (b). However, the latter goes at the expense of the signal-to-noise ratio, as seen from the large scatter of the data for a relative intensity of 0.1 (red line). This translates to a lower spatial resolution as the resolution is limited by the maximum scattering angle at which diffraction data are recorded with sufficient statistics. Similarly, by moving the detector further back, not only are the scattered photons dispersed over more pixels, which increases the recall, but also the large-angle scattering signal becomes too sparse, and the maximum usable scattering angle is reduced.

When we replot all the data shown in (a) and (b) as a function of the area density of scattered photons on the detector, i.e., the mean number of photons per pixel, all curves overlap almost perfectly, see Supplementary Fig. 5c. Thus, the photon density is the decisive quantity that should be kept below 0.1 as the recall steeply drops to zero above this value.

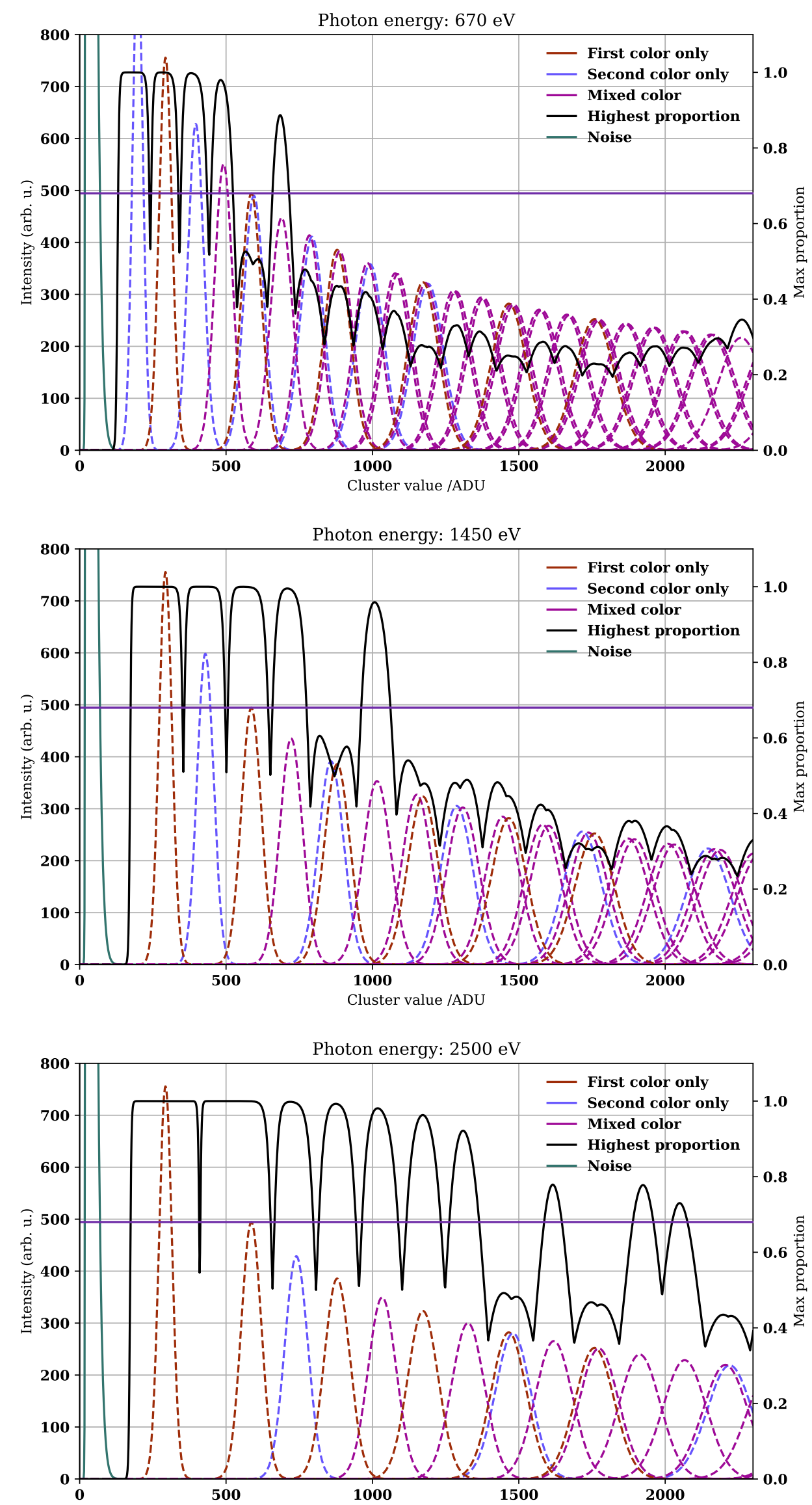

Supplementary Fig. 6. Simulated distributions of pixel values as shown and explained in Fig. 8 (in the Methods section) for the combination of photon energies $E_{\mathrm{ph},1} = 1000$ eV and $E_{\mathrm{ph},2} = 670$, 1450, and 2500 eV.

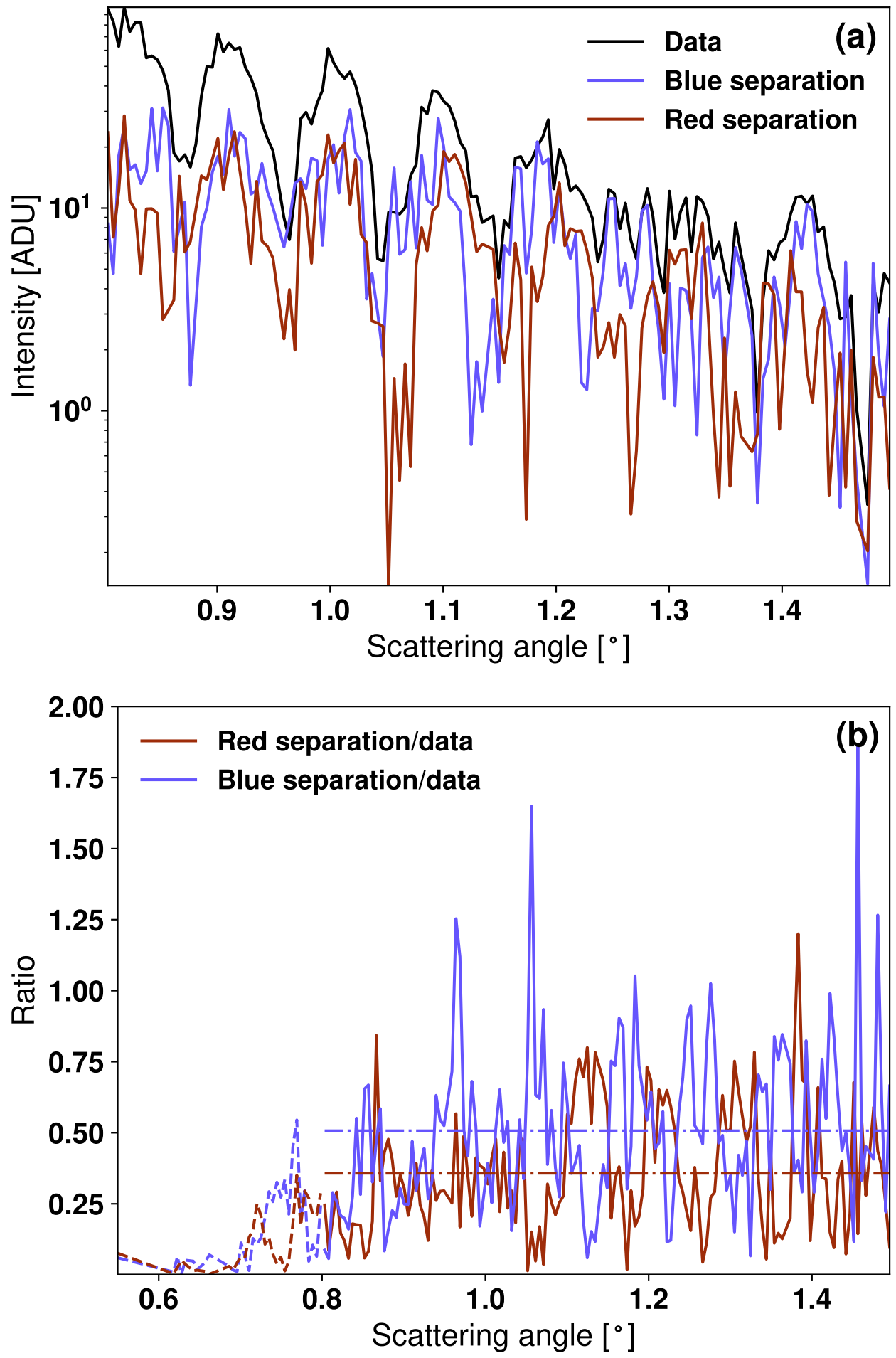

Supplementary Fig. 7. (a) Radial intensity profiles of a selected diffraction pattern where the fluence of red and blue X-ray pulses was approximately equal. The black line is the profile of the total two-color pattern, the red and blue lines are the profiles of the pixel-based color-separated patterns. (b) Ratio of radial intensities of color-separated patterns with respect to the full pattern. At scattering angles $< 0.8^\circ$ (dashed part), the color separation is inefficient, whereas at angles $> 0.8^\circ$, average relative intensities of 0.36/0.51 for the red/blue pulse are reached, marked with the horizontal lines.

Supplementary Table 1. Overview of single photon patterns found in the brightest 75 shots. Naming as in Supplementary Fig. 9.

Shape	Fraction [%]
single	46
horizontal	21
vertical	17
fuL	3
uL	3
L	3
fL	3
quadrant	4

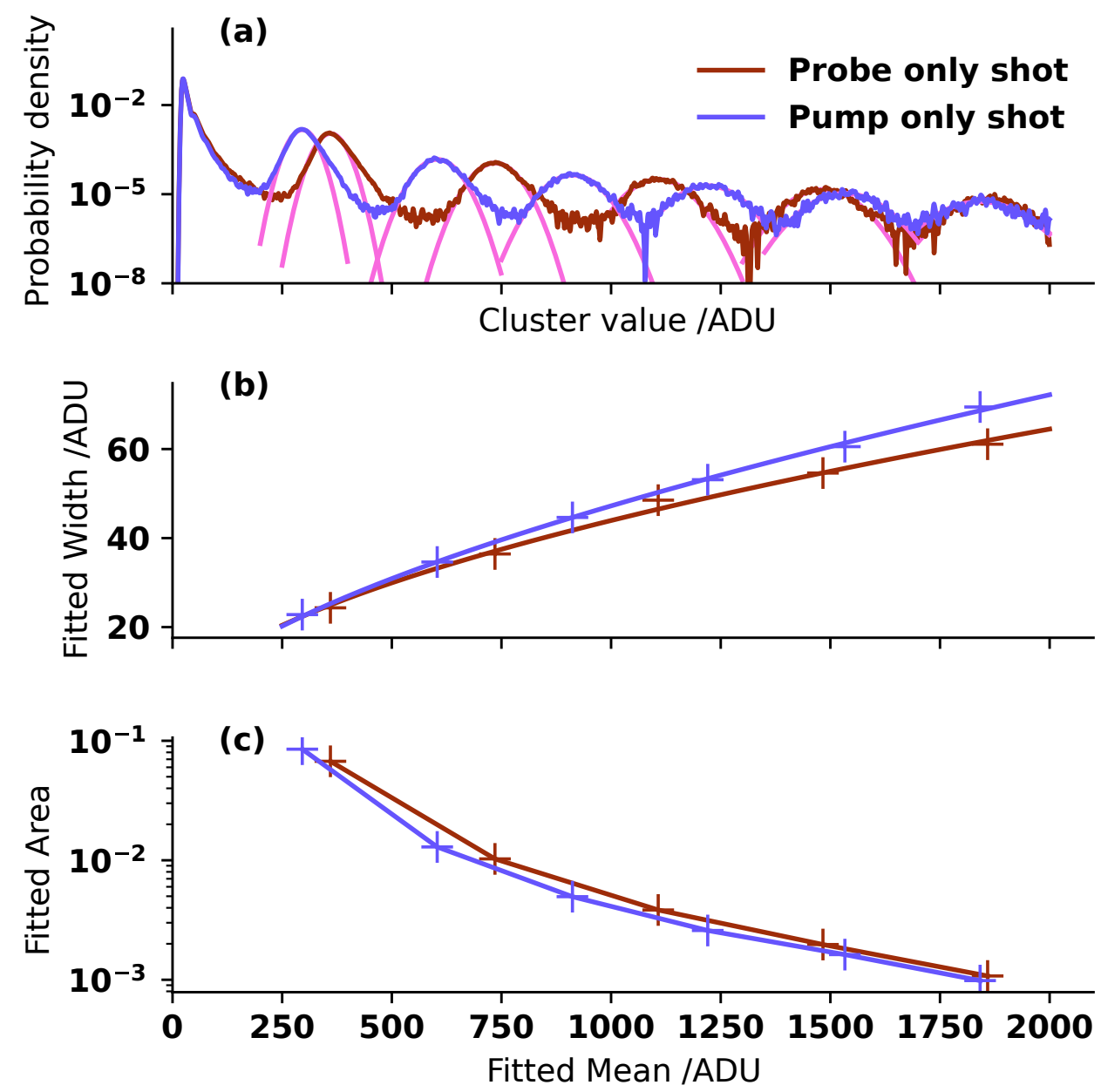

Supplementary Fig. 8. Analysis of the distribution of pixel counts for a set of measured patterns recorded using “blue pulses” only. The areas of the one-photon, two-photon etc. peaks in the histograms in (a) quickly drop as a function of the number of photons, (c). The widths of the peaks increase with the intensity as $\bar{I}^{0.55}$ for the red and as $\bar{I}^{0.6}$ for the blue pulses, (b).

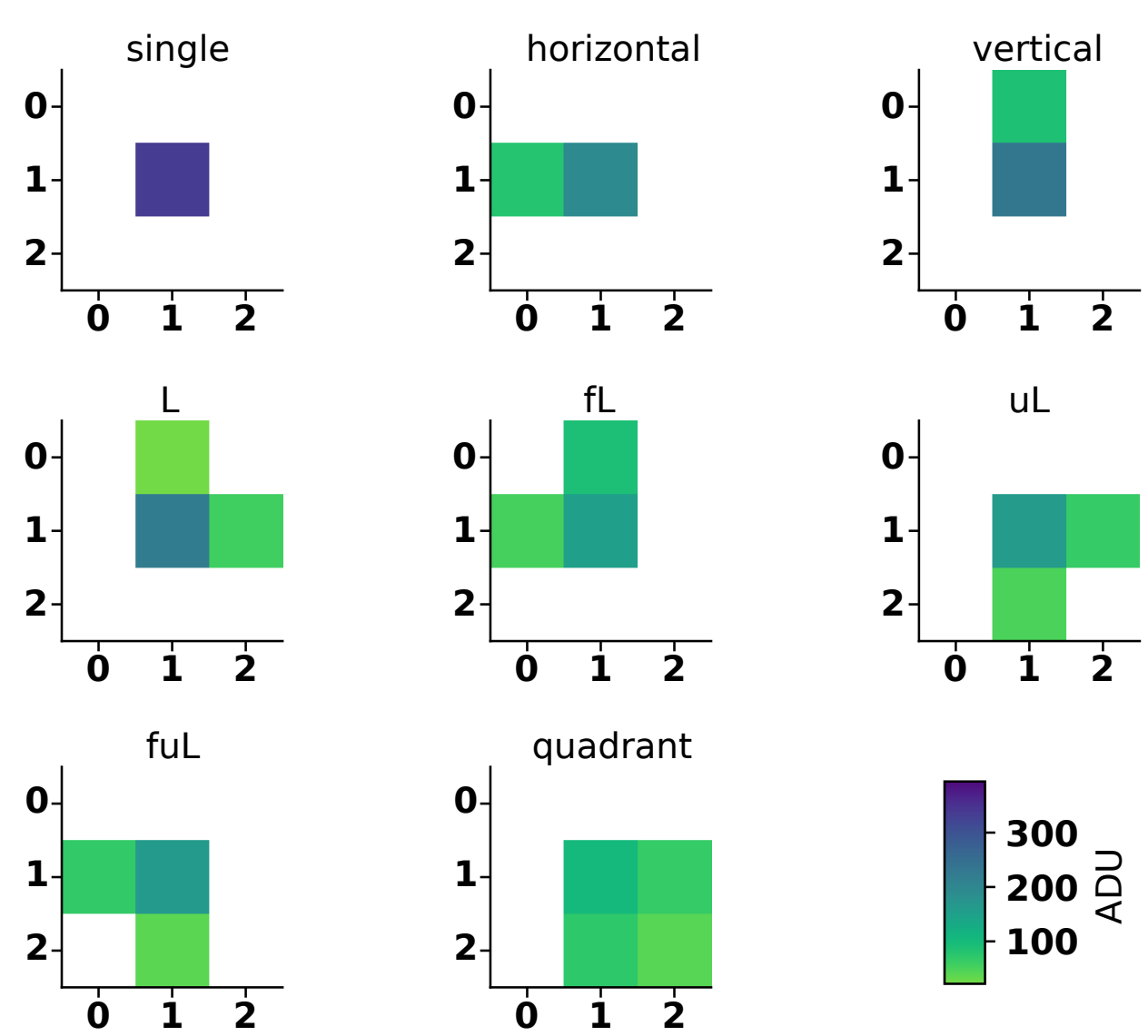

Supplementary Fig. 9. All possible pixel patterns produced by a single photon.

Bounding the positional shift in cluster assignment

Assigning a cluster places it at its center-of-mass pixel. While this introduces a slight coordinate shift in the diffraction pattern, this effect is limited since the procedure is only feasible for small, few-photon clusters. To determine an upper bound for this shift, we calculated the Euclidean distance from the center-of-mass pixel to the furthest extent of the original pattern. These are shown in Supplementary Fig. 10 for 10 randomly chosen diffraction patterns processed with 50% confidence. As shown, for the vast majority of patterns, the actual photon impact point is shifted by at most $\sqrt{2}$ pixels from the assigned pixel coordinate. This results in an average shift of 0.8 pixels using this conservative upper bound estimate.

Impact of positional uncertainty on structural resolution

Our assignment procedure introduces a localized shift of approximately 0.8 pixels. In this section, we briefly discuss how this uncertainty in photon position does not affect the information content of the diffraction data. The discussion involves three aspects: signal oversampling, geometric error scaling, and physical bandwidth limits.

In single-particle CDI, the maximum frequency of signal variation on the detector is strictly constrained by the sample's spatial extent. The Fourier transform of the diffraction pattern corresponds to the sample's autocorrelation function. For an isolated sample of radius R, the autocorrelation has a maximum extension of $D_{\max} = 4R$. Consequently, the fastest oscillation (fringe period) in the diffraction signal occurs over a reciprocal length $\Delta q = \frac{\pi}{R}$.

In our experimental geometry ($\lambda = 1.24\,\mathrm{nm}$, pixel-size $\delta\theta = 0.0126\,^\circ$, this characteristic length can be expressed in pixels (M) for the average-sized particle used in the main text and the maximum-sized particle detected:

$$M(354\,\mathrm{nm}) = 8.0 \text{ pixels} \tag{6}$$

$$M(850\,\mathrm{nm}) = 3.3 \text{ pixels} \tag{7}$$

A positional uncertainty of 1–2 pixels acts as a point spread function (PSF) that is significantly narrower than the minimum oscillation period of the signal. Thus, this localized shift preserves the structural information without significant blurring.

Furthermore, the sensitivity of real-space structural features to reciprocal-space uncertainties decreases at higher scattering angles. For a feature of size x associated with a momentum transfer q, the relationship $x = \frac{2\pi}{q}$ implies, via error propagation, that the real-space uncertainty Δx scales as:

$$\Delta x = \left| \frac{2\pi}{q^2} \right| \Delta q \tag{8}$$

For a fixed detector pixel uncertainty Δq, the resulting error in the estimated structural size Δx is reduced by a factor of q^2. For example, a one-pixel error at a scattering angle of $0.3\,^\circ$ corresponds to an uncertainty of $\sim 10\,\mathrm{nm}$, whereas the same error at $3\,^\circ$ (high-q) corresponds to only $\sim 1\,\text{Å}$. Thus, the high-resolution region of the detector is inherently more robust to absolute pixel misplacements.

On top of these purely mathematical considerations, the $1\,\%$ bandwidth of the FEL introduces an intrinsic coordinate jitter in the scattering angle. At high scattering angles (e.g., $1.5\,^\circ$), this jitter is ~ 1 pixel, making the cluster-assignment shift commensurate with the existing physical limits of the experiment.

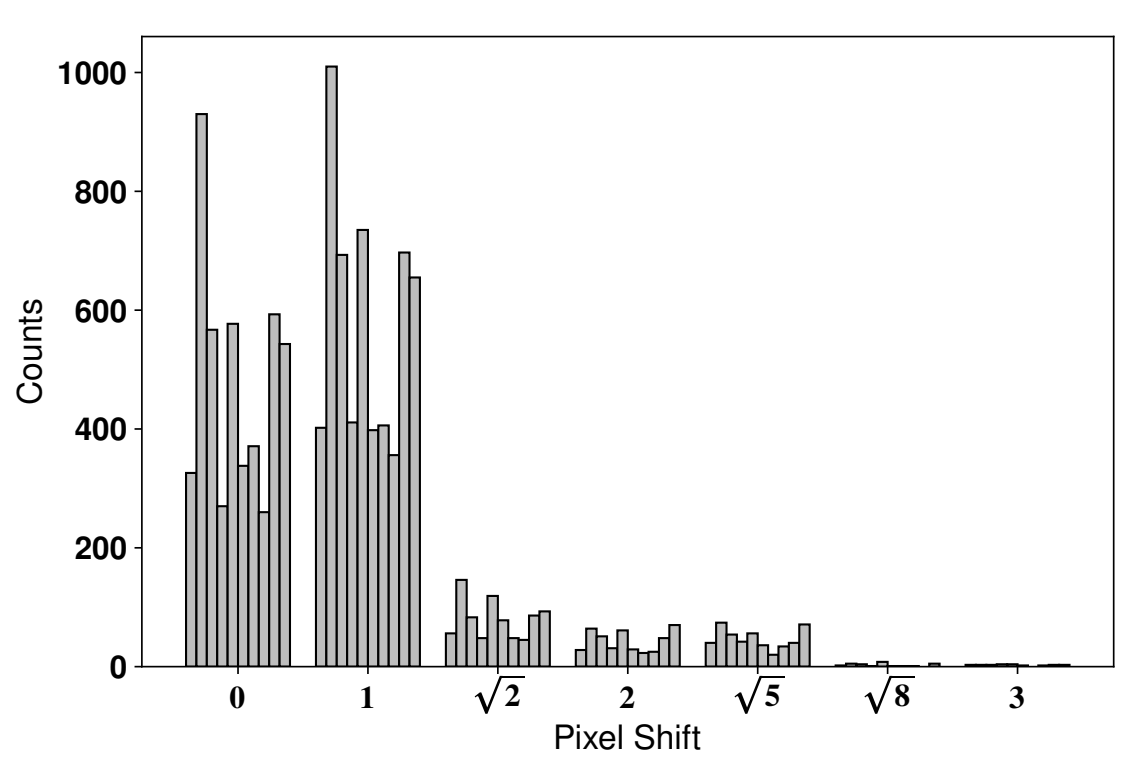

Supplementary Fig. 10. Upper bound on the distance between the edge of a photon cluster and its assigned pixel position. Ten randomly selected diffraction patterns were analyzed.

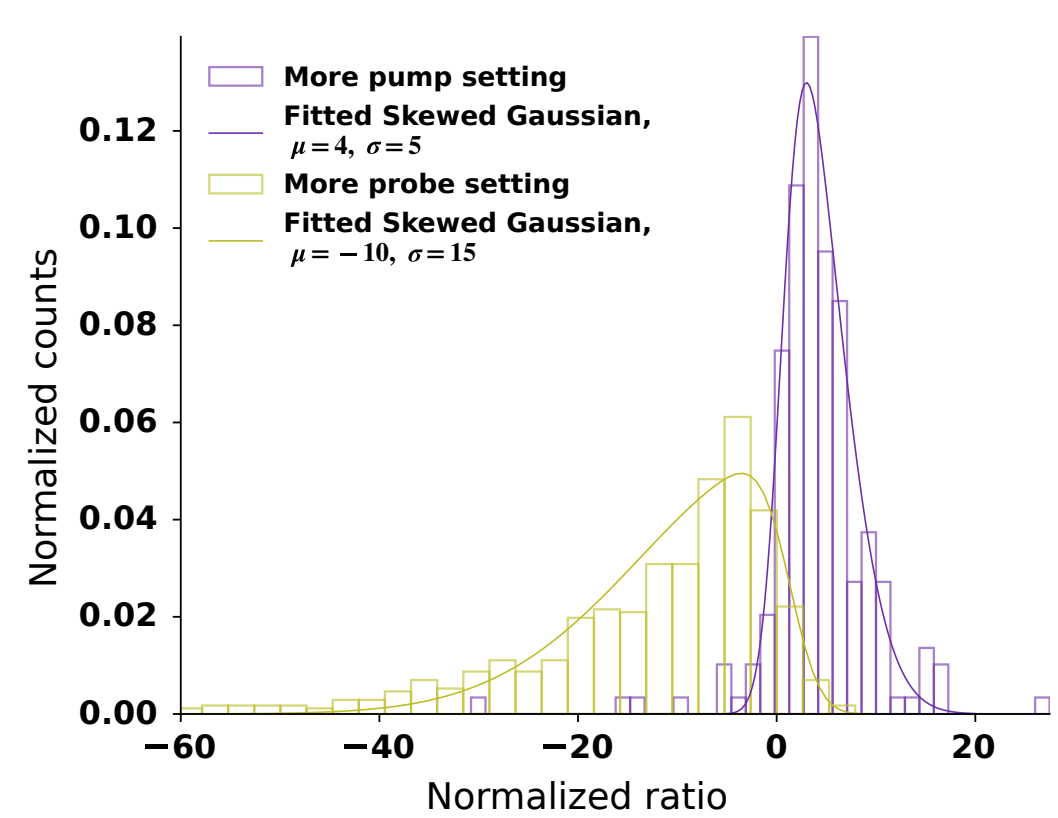

Supplementary Fig. 11. Abundance distributions of the ratios of intensities of the two x-ray pulses, $I_{\text{red, blue}}$ as determined from the pixel-based separation of patterns. The positive side of the x-axis shows the abundance of ratios $I_{\text{red}}/I_{\text{blue}} - 1$ where $I_{\text{red}}/I_{\text{blue}} > 1$, the negative side shows $1 - I_{\text{blue}}/I_{\text{red}}$ where $I_{\text{red}}/I_{\text{blue}} < 1$. At $x = 0$, $I_{\text{red}}/I_{\text{blue}} = 1$. The data are fitted with a skewed Gaussian function, with μ describing the position of the distribution and σ its characteristic width.
