# Supplementary material for: Model-free pattern separation of two-color ultrafast X-ray diffraction
Source: Nat Commun. 2026 Sep 3;17:10465. doi: 10.1038/s41467-026-77085-z (PMC13635185; doi:10.1038/s41467-026-77085-z)
Supplement: Supplementary file 2 [file 41467_2026_77085_MOESM2_ESM.pdf]

Peer Review File

Model-free pattern separation of two-color ultrafast X-ray diffraction

Corresponding Author: Mr Linos Hecht

This file contains all reviewer reports in order by version, followed by all author rebuttals in order by version.

Version 0:

Reviewer comments:

Reviewer #1

(Remarks to the Author)
The manuscript presents a coherent diffraction imaging experiment on helium nanodroplets using two hard X-ray pulses with different photon energies, separated in time by approximately 750 femtoseconds. The novelty of this work lies in a pixel-based color separation method applied to distinguish the diffraction patterns resulting from the two pulses, separating them into two individual diffraction patterns corresponding to each pulse. This is possible because the ADU readings of single-photon detector pixels depend on photon energy, enabling the determination of both the number and corresponding energies of photons detected by each pixel. I find this work quite interesting, as it provides a new direction in ultrafast X-ray diffraction experiments.

I have two main concerns before considering its acceptance:

1. The technique is demonstrated on spherical helium nanodroplets under the assumption that the particle does not interact with the first X-ray pulse. While I like the idea of separating diffraction patterns from two X-ray pulses, it would be helpful to elaborate more on the limitations of the method. For instance, would this approach still work if the first X-ray pulse induces ultrafast dynamics or damage in the sample? If yes, is there an adaptive procedure that needs to be implemented? Furthermore, what is the broader applicability of this technique beyond non-interacting spherical nanodroplets?

2. I believe the current pixel-based classification assumes that the main diffraction signal is elastic. For samples exhibiting strong inelastic scattering, the scattered photons would have different energies compared to the incident X-rays. Is there a proposed solution or adjustment for this scenario?

Minor comments:
1. Page 2, line 140: It should be 4.2×10^15 W/cm^2, otherwise it currently reads as 4.2×10^16 W/cm^2.

2. Page 2, line 172: the dashed pink line in the inset of Figure 1a is not visible.

Reviewer #2

(Remarks to the Author)
The paper of Hecht et. al. reports the use of two time-delayed X-ray pulses of different energies to study ultrafast structural changes in nanoscale particles. The authors sought to demonstrate that, by separating overlapping diffraction images with energy-sensitive detection and pattern analysis, the method is validated against Mie modeling and has a strong potential for advanced X-ray ultrafast imaging. In the XFEL experiment, although the time delay between X-ray pulses can be adjusted on the femtosecond scale, the individual pulses cannot be distinguished at the detector due to the slow shutter time. We found the approach presented in this paper to be an interesting way to overcome this limitation, and the pixel-based color-separation method appears promising. However, several issues should be addressed. I list questions and suggestions below.

1. The title of the manuscript includes Coherent Diffraction Imaging (CDI). However, the content primarily focuses on the successful separation of two-color pixel-based X-ray “patterns”. The authors seem to have misused the terms “Coherent Diffraction Pattern” and “Coherent Diffraction Imaging”.

2. If this work is intended to be related to CDI, it should be clarified. CDI is based on the phase retrieval algorithms, and its accuracy depends on maintaining consistency in the retrieved phase. High-intensity regions are more likely to yield successful phase retrieval. In contrast, the primary analysis in this paper focuses on regions corresponding to approximately 0.1 detected photons per pixel, which represents a very low-intensity regime. While the demonstration of two-color X-ray separation under these conditions is interesting, the connection of this work to CDI appears relatively weak, and the scope may not be appropriate.
3. In Fig. 1, the main image had an inset showing the radial intensity profile, which is described as the result of angular integration (as stated in the caption and in line 151). However, the detector image contains masked regions to prevent proper radial integration. If the radial integration was performed simply by summing pixel values, the resulting profile would not accurately represent the true scattering signal. If instead the values were normalized on a per-pixel basis (i.e., averaged), this should be explicitly stated. A clear description of how the radial profile was constructed is needed.
4. In Fig. 5(b), the black curve represents the combined red and blue signal, yet it lies below the individual red and blue curves. Was the scale adjusted independently? Some explanation for this discrepancy is necessary.
5. In Fig. 6(b), single- and multi-photon events are classified based on Fig. 6(a). In Fig. 6(c), the two-color separation results are presented. However, some pixel information that appears in (b) is missing in (c). This may be related to the merging of spill-over effects, and requires further classification. In addition, the example in panels (8,10) raises a question: in (a) and (b), five connected pixels appear to form an overlapped an overlapped “fuL” pattern, but they are assigned as a two-photon event. How was this assignment determined at the pixel level?
6. Regarding the probabilities, the ADU distributions for red, blue, and mixed-color cases are shown in Fig. 7. For the single-photon case, red and blue photons appear to be well separated. In the multi-photon case, however, the probability of mixed-color events is treated as a simple linear relation. In probability theory, hthe likelihood of obtaining a mixed-color event should generally exceed that of purely red–red or blue–blue events, assuming independent events. When deriving multi-photon probabilities from single-photon probabilities, the binomial coefficient should be included. Therefore, the analyses may need to be repeated.
7. In Fig. 9(b), the photon energy was scaled by a factor of 0.05 to match the scattering angle range to the experimental data due to computational limitations in MD simulations. This adjustment seems somewhat artificial and warrants further justification.
8. The Supplementary Figures (Fig. SMs) are not in proper order. For example, the first supplementary figure referred at line 136 is Fig. SM7. Additionally, a caption of Fig. SM8, referenced at line 376, refers to Fig. 7, although only Fig. 3 has been introduced at that point.

Reviewer #3

(Remarks to the Author)
I co-reviewed this manuscript with one of the reviewers who provided the listed reports. This is part of the Nature Communications initiative to facilitate training in peer review and to provide appropriate recognition for Early Career Researchers who co-review manuscripts.

Version 1:

Reviewer comments:

Reviewer #1

(Remarks to the Author)
All my previous comments have been addressed satisfactorily, and the paper can be accepted from my perspective.

Reviewer #2

(Remarks to the Author)
I thank the authors for their careful and detailed responses to the original comments. Overall, the manuscript has been substantially improved, and most of my concerns have been satisfactorily addressed. Two minor points remain on #5, which I would ask the authors to clarify before publication.
The authors' explanation of the cluster-collapsing algorithm — in which a connected cluster is identified, its total ADU value is summed, the number and color composition of photons is determined at the cluster level, and the result is collapsed onto the pixel nearest the cluster's center of mass — clearly answers how the two-photon event at (8,10) is assigned. The correction of the certainty-threshold inconsistency (from 65% back to 50%) is also appropriate. On this primary point, the response is satisfactory.
Two concerns remain, however, that I would like the authors to address:
(a) Potential internal tension between the arguments in Comments 2 and 5. In their response to #2, the authors argue that clean wavelength separation at high-q will strongly benefit imaging and will help determine accurate structural features within a multi-wavelength phase-retrieval framework. In the present response, however, the 1–2 pixel displacement introduced by the center-of-mass assignment is justified by the statement that "a non-systematic displacement of 1–2 pixels in the detector plane does not measurably alter the scattering angles or the structural features extracted during the subsequent CDI reconstruction." These two positions appear to be in tension: if high-q separation is critical for resolution, a

1–2 pixel positional error in exactly that region cannot be dismissed as negligible. I ask the authors to reconcile these two statements explicitly in the revised text — for instance, by quantifying the regime in which the 1–2 pixel shift is indeed negligible, and by clarifying where and how this condition is compatible with the resolution benefits claimed in Comment 2.
(b) Inconsistency between the response letter and the revised manuscript. The authors quote the following sentence as having been inserted into the main text:
"Clearly, the number of illuminated pixels is significantly reduced in the color-separated images. First, ADU values are discarded if they are classified as noise or if their assignment to integer multiples of the red or blue elementary ADU values is ambiguous. Second, clusters of illuminated pixels are collapsed into the pixel position closest to the cluster's center of mass (see Methods for details)."
In the revised manuscript (around line 256), the inserted wording conveys a similar meaning but does not precisely match the text given in the response letter. Please verify that the response letter and the final manuscript are fully consistent.

Reviewer #3

(Remarks to the Author)
I co-reviewed this manuscript with one of the reviewers who provided the listed reports. This is part of the Nature Communications initiative to facilitate training in peer review and to provide appropriate recognition for Early Career Researchers who co-review manuscripts.

Version 2:

Reviewer comments:

Reviewer #2

(Remarks to the Author)
Both of the remaining points are appropriately reflected in the uploaded manuscript. For (a), both the promised main-text paragraph (lines 635–646) and the quantitative analysis section in the Supporting Information are present. For (b), the main-text wording matches the final version confirmed in the rebuttal. Both items appear consistent and require no further revision.

Reviewer #3

(Remarks to the Author)
I co-reviewed this manuscript with one of the reviewers who provided the listed reports. This is part of the Nature Communications initiative to facilitate training in peer review and to provide appropriate recognition for Early Career Researchers who co-review manuscripts.

Open Access This Peer Review File is licensed under a Creative Commons Attribution 4.0 International License, which permits use, sharing, adaptation, distribution and reproduction in any medium or format, as long as you give appropriate credit to the original author(s) and the source, provide a link to the Creative Commons license, and indicate if changes were made.

In cases where reviewers are anonymous, credit should be given to 'Anonymous Referee' and the source.
The images or other third party material in this Peer Review File are included in the article's Creative Commons license, unless indicated otherwise in a credit line to the material. If material is not included in the article's Creative Commons license and your intended use is not permitted by statutory regulation or exceeds the permitted use, you will need to obtain permission directly from the copyright holder.
To view a copy of this license, visit https://creativecommons.org/licenses/by/4.0/

Response to reviews on manuscript NCOMMS-25-78444-T.

We sincerely thank the Editor and the Reviewers for their careful evaluation of our manuscript and for providing constructive feedback. In the revised version, we have addressed all comments, resulting in a manuscript with significantly improved clarity and rigor. We hope that these revisions have resolved the concerns raised and that the manuscript is now suitable for publication. Below, we provide a point-by-point response to each comment, along with the corresponding changes in the revised manuscript, which are highlighted in blue.

REVIEWER COMMENTS

Reviewer #1 (Remarks to the Author):

> *The manuscript presents a coherent diffraction imaging experiment on helium nanodroplets using two hard X-ray pulses with different photon energies, separated in time by approximately 750 femtoseconds. The novelty of this work lies in a pixel-based color separation method applied to distinguish the diffraction patterns resulting from the two pulses, separating them into two individual diffraction patterns corresponding to each pulse. This is possible because the ADU readings of single-photon detector pixels depend on photon energy, enabling the determination of both the number and corresponding energies of photons detected by each pixel. I find this work quite interesting, as it provides a new direction in ultrafast X-ray diffraction experiments.*

We thank the Reviewer for their positive evaluation, in particular for acknowledging the novelty of our work.

> *I have two main concerns before considering its acceptance:*
>
> *1. The technique is demonstrated on spherical helium nanodroplets under the assumption that the particle does not interact with the first X-ray pulse. While I like the idea of separating diffraction patterns from two X-ray pulses, it would be helpful to elaborate more on the limitations of the method. For instance, would this approach still work if the first X-ray pulse induces ultrafast dynamics or damage in the sample? If yes, is there an adaptive procedure that needs to be implemented? Furthermore, what is the broader applicability of this technique beyond non-interacting spherical nanodroplets?*

The Reviewer raises an important point about the method's applicability in the presence of ultrafast dynamics or structural damage and its broader applicability. Indeed, enabling such dynamic experiments is the main motivation for developing this two-color image-separation approach. We have revised the introduction of our manuscript to clarify this aspect. In particular, we envision two experimental scenarios:

(i) **X-ray–pump / X-ray–probe experiments**, in which the first FEL pulse initiates structural dynamics in a single nanoparticle while simultaneously providing a snapshot of its initial state (size, shape, orientation, illumination intensity). The second pulse probes the evolution of the structure at a controlled delay, enabling time-resolved structural reconstruction and ultimately the creation of an X-ray-induced structural-dynamics “movie” of nanoscale objects in the gas phase.

(ii) **Probe–pump–probe schemes**, where the first X-ray pulse records the initial state with minimal perturbation, an intense optical laser pulse induces the dominant dynamics, and the second X-ray pulse probes the transient structure. This approach is especially attractive for studying nanoplasma dynamics, such as shock waves and other types of laser-driven nonequilibrium states in nanomatter.

In both scenarios, reliable separation of diffraction images from two X-ray pulses is a prerequisite for capturing the initial and time-evolved states of the nanoparticle. The present work establishes this capability under controlled conditions. Our current experiment basically represents the demonstration of the second approach, but without the presence of a "pump" pulse driving strong dynamics. Despite the 750 fs delay between the two probe pulses, the recorded patterns show no structural changes of the irradiated nanodroplets within the resolution of our experiment.

This aspect is essential for rigorous benchmarking of our method before applying it to dynamically evolving targets. By choosing pristine helium nanodroplets and photon energies in the vicinity of 1 keV, we kept ultrafast structural dynamics far below the spatial resolution of our experiment. Separating the diffraction signal of the two superimposed patterns via their intensity values relies solely on the difference in photon energies; it doesn't involve any model assumptions for the initial state, stays valid for all possible structural changes between the pump and probe pulses, and is generally applicable to diffraction methods of all kinds. In the present case of helium nanodroplets irradiated by relatively weak X-ray pulses, independent validation of these results is uniquely possible because we can constrain the two-color Mie simulations to the same spherical object.

Concerning the Reviewer's question whether method adaptations are required in the presence of structural dynamics in the target: as long as the detector signal consists of elastically scattered photons from the two pulses (for more details on this point, see our response to the second comment), the pixel-based color separation works as presented here. Structural evolution (e.g., expansion, density redistribution) would modify the diffraction pattern, but not impair the image-separation method itself, as it is valid for all shapes and structures. A validation of the separated patterns by Mie fitting is, of course, limited to spherical shapes, but provided the change of the target structure is spherically symmetric, the two-color Mie-fitting approach could be extended to more complex radial density profiles (Peltz 2022).

At the current stage, the color-separation method should be viewed as a first but essential step rather than a complete solution to the two-pulse single-particle coherent diffraction imaging problem. The main limitation of the method is its restriction to detector regions with photon densities below ~0.1 photons per pixel. At low momentum transfer (low *q*), spectral pile-up prevents reliable discrimination of the two color contributions. However, as single-particle coherent X-ray diffraction is strongly peaked in the forward direction, only a few photons hit the detector in high-*q* regions. Therefore, these regions, where the finer structural details are encoded, are intrinsically favored for pixel-based color separation. In this regard, the color separation method can serve as an essential building block towards two-color time-dependent structure determination within two-wavelength phase-retrieval frameworks, as it provides additional constraints for iterative algorithms.

Even beyond single-particle CDI, it is perfectly applicable to all kinds of X-ray diffraction methods when extended to two-color illumination for dynamic investigations.

In summary, in the revised version of the manuscript, we describe envisioned applications for which a pixel-based color-separation method is required, as well as the clear limitations of our approach. In addition, we have extended the discussion section with an overview of possible extensions to our approach.

> *2. I believe the current pixel-based classification assumes that the main diffraction signal is elastic. For samples exhibiting strong inelastic scattering, the scattered photons would have different energies compared to the incident X-rays. Is there a proposed solution or adjustment for this scenario?*

The pixel-based classification as presented in this work indeed assumes that the detector signal arises from elastic scattering and thus contains the contribution of two photon energies. This assumption is appropriate as in the present experiment, the photon energies are far from any electronic resonances of helium, and inelastic scattering (e.g., X-ray Raman, Compton scattering) and contributions from other incoherent processes (e.g., fluorescence) are negligible.

Under resonant excitation or in strongly driven systems, however, nonlinear population dynamics, fluorescence, stimulated emission, or other inelastic contributions may become significant [1-4]. In particular, if a nanoplasma is generated by the interaction with the X-ray pulse, both XUV and X-ray line emission (bound-bound) and continuum emission (free-bound and free-free) can occur. In such cases, photons with different energies can indeed contribute to the detected signal. In principle, our color-separation method is well suited to discriminate photons of different energies, provided that the energy differences exceed the detector's spectral resolution limit (≈10% for the pnCCD detector used here). Fluorescence photons, for example, could therefore be separated if their energies are sufficiently distinct from the elastically scattered photons. Small energy shifts arising from ultrafast electronic dynamics, however, would likely remain unresolved. Algorithmically, extending the approach to include an additional photon energy (“third color”) would be straightforward. Corresponding limitations are not conceptual but set by detector pixel size, energy resolution, and photon statistics.

We have added an explicit statement in the results section of the revised manuscript about our assumption of elastic scattering (lines 164 ff.) and a more extensive explanation of the appropriateness of this assumption in the methods section (lines 512 ff.). In addition, the modified discussion now also contains the discussion of additional photon energies in the detector signal as a consequence of X-ray- or laser-matter interaction and the possible implementation of those into the pixel-wise separation method (lines 438 ff.).

[1] Robert G. Radloff, Nils Muthreich, Felix Zimmermann, Yanwen Sun, Taito Osaka, Gota Yamaguchi, Jumpei Yamada, Anatoli Ulmer, Slawomir Skruszewicz, Phay J. Ho, Ichiro Inoue, Tais Gorkhover, and Stephan Kuschel “Enhancement of scattering and absorption cross-sections by transient resonances in copper and iron”, Proc. SPIE PC13536, X-Ray Free-Electron Lasers: Advances in Source Development and Instrumentation VII, PC135360Z (6 June 2025), https://doi.org/10.1117/12.3058359

[2] Stephan Kuschel, Phay J. Ho, Andre Al Haddad, Felix F. Zimmermann, Leonie Flueckiger, Matthew R. Ware, Joseph Duris, James P. MacArthur, AlbertoLutman, Ming-Fu Lin, Xiang Li, Kazutaka Nakahara, Jeff W. Aldrich, Peter Walter, Linda Young, Christoph Bostedt, Agostino Marinelli, and Tais Gorkhover "Non-linear enhancement of ultrafast X-ray diffraction through transient resonances", *Nature Communications*, **16**, 847 (2025), https://doi.org/10.21203/rs.3.rs-3776765/v1

[3] Robert G. Radloff, Felix F. Zimmermann, Siqi Li, Stephan Kuschel, Anatoli Ulmer, Yanwen Sun, Takahiro Sato, Peihao Sun, Johann Haber, Diling Zhu, Miklós Tegze, Gyula Faigel, Matthew R. Ware, Jordan T. O'Neal, Jumpei Yamada, Taito Osaka, Robert Zierold, Carina Hedrich, Dimitrios Kazazis, Yasin Ekinci, Makina Yabashi, Ichiro Inoue, Andrew Aquila, Meng Liang, Agostino Marinelli, Tais Gorkhover "Fluorescence intensity correlations enable 3D imaging without sample rotations", arXiv:2510.24386v2

[4] Björn Kruse, Benjamin Liewehr, Christian Peltz, and Thomas Fennel "Quantum coherent diffractive imaging", J. Phys. Photonics **2** (2020) 024007, https://doi.org/10.1088/2515-7647/ab83e4

Minor comments:
1. Page 2, line 140: It should be 4.2×10^15 W/cm^2, otherwise it currently reads as 4.2×10^16 W/cm^2.
2. Page 2, line 172: the dashed pink line in the inset of Figure 1a is not visible.

We thank the Reviewer for catching these mistakes. We have corrected them in the revised manuscript.

Reviewer #2 (Remarks to the Author):

The paper of Hecht et. al. reports the use of two time-delayed X-ray pulses of different energies to study ultrafast structural changes in nanoscale particles. The authors sought to demonstrate that, by separating overlapping diffraction images with energy-sensitive detection and pattern analysis, the method is validated against Mie modeling and has a strong potential for advanced X-ray ultrafast imaging. In the XFEL experiment, although the time delay between X-ray pulses can be adjusted on the femtosecond scale, the individual pulses cannot be distinguished at the detector due to the slow shutter time. We found the approach presented in this paper to be an interesting way to overcome this limitation, and the pixel-based color-separation method appears promising.

We are grateful for the Reviewer's positive reception of our work and particularly appreciate their recognition of the future potential of the color separation method.

However, several issues should be addressed. I list questions and suggestions below.
1. The title of the manuscript includes Coherent Diffraction Imaging (CDI). However, the content primarily focuses on the successful separation of two-color pixel-based X-ray "patterns". The authors seem to have misused the terms "Coherent Diffraction Pattern" and "Coherent Diffraction Imaging".

We agree with the Reviewer that the manuscript focuses on the separation of diffraction patterns rather than on the aspect of imaging. The revised title now reads *"Model-free*

pattern separation of two-color ultrafast X-ray diffraction". The wording in the revised manuscript was changed from *"diffraction images"* to *"diffraction patterns"* for consistency.

2. If this work is intended to be related to CDI, it should be clarified. CDI is based on the phase retrieval algorithms, and its accuracy depends on maintaining consistency in the retrieved phase. High-intensity regions are more likely to yield successful phase retrieval. In contrast, the primary analysis in this paper focuses on regions corresponding to approximately 0.1 detected photons per pixel, which represents a very low-intensity regime. While the demonstration of two-color X-ray separation under these conditions is interesting, the connection of this work to CDI appears relatively weak, and the scope may not be appropriate.

This question has already been partially addressed in our response to the first comment of Reviewer 2 and the first and second comments of Reviewer 1. In addition, we note that for the particular case of ideally spherical helium droplets, the two-color Mie fitting does indeed provide an appropriate way to extract spatial and electronic properties of the target, which, in our view, fulfills the requirements of an "imaging method". The pixel-based color separation, the main finding of our work and a more broadly applicable approach, is restricted to detector regions where the photon count rate remains below approximately 0.1 photons per pixel, limiting it to high-*q* regions in single-particle coherent diffraction experiments. In this sense, the pixel-based separation method alone does not yet meet the requirements of fully time-resolved single-particle coherent diffraction imaging (CDI), where access to information across the entire detector area for both colors is essential.

However, clean wavelength separation at high *q* will strongly benefit "true imaging methods". It can provide reliable boundary conditions in pattern regions where, e.g., phase retrieval methods lack accuracy. This helps determine accurate pulse intensities for both colors and, ultimately, enables time-dependent final structure determination within multi-wavelength phase-retrieval frameworks. Our long-term strategy for further developing the pixel-based separation method also includes extending the approach to higher photon-density regimes. We envision the development of a multi-wavelength phase-retrieval framework directly based on the pixel-wise separation method using cross-correlation maps of pump-only and mixed pump–probe diffraction patterns, akin to the method described in [1], which could allow the extraction of structural dynamics information.

[1] Tim B. Berberich, Serguei L. Molodtsov and Ruslan P. Kurta, "A workflow for single-particle structure determination via iterative phasing of rotational invariants in fluctuation X-ray scattering", J. Appl. Crystallogr. 57, 324-343, https://doi.org/10.1107/S1600576724000992

In addition to changing the title and the wording from "imaging" to "pattern", we have revised the introduction and discussion sections to explore possible future extensions of our approach and its potential impact on various kinds of diffraction-based methods (see lines 140 ff. and 414 ff.).

3. In Fig. 1, the main image had an inset showing the radial intensity profile, which is described as the result of angular integration (as stated in the caption and in line 151). However, the detector image contains masked regions to prevent proper radial

integration. If the radial integration was performed simply by summing pixel values, the resulting profile would not accurately represent the true scattering signal. If instead the values were normalized on a per-pixel basis (i.e., averaged), this should be explicitly stated. A clear description of how the radial profile was constructed is needed.

We thank the Reviewer for pointing out this important detail. We realize that our use of the term 'integrated' in the original manuscript was imprecise, as it could imply a simple summation of pixel values. In fact, our procedure always involved azimuthal averaging to ensure that masked regions do not bias the radial profile. We have corrected the terminology throughout the revised manuscript to accurately reflect this per-pixel normalization. We added a clearer explanation of how the radial profiles are obtained in line 181:

"It is obtained by angular (azimuthal) averaging based on the count of non-masked pixels within each radial bin. In this way, masked pixels do not bias the radial intensity, though their exclusion slightly reduces the signal-to-noise ratio."

Additionally, we clarified what is shown in Fig. 1 (b) and in the inset in Fig. 1 (a).

"Fig. 1 (b) shows a fit of the radial intensity distribution of a bichromatic field scattered off a hard sphere [44,45] (pink dashed line) to the experimentally measured radial profile (black line). It is a magnified view of the radial profile shown in the inset of Fig. 1 (a). The solid red and blue lines represent the individual color contributions that add up to the bichromatic fit shown by the dashed pink line. More examples of such radial profiles and fits are given in Figs. SM2 and SM3 ."

4. In Fig. 5(b), the black curve represents the combined red and blue signal, yet it lies below the individual red and blue curves. Was the scale adjusted independently? Some explanation for this discrepancy is necessary.

The three curves represent histograms of three single shots from different experimental configurations, e.g., independent measurements performed with different undulator settings. Specifically, the black curve corresponds to the single-shot data shown in Fig. 1 for a two-color configuration. The other two "red/blue only" curves represent single-shot data, taken from different single-color configurations. All shown data are raw counts; no rescaling was performed.

The selection of the individual shots was done such that the intensities are roughly comparable. This subfigure primarily emphasizes that the assignment of peak positions matches the individual colors and that the detector resolution is sufficient to distinguish them in the two-color case. Furthermore, by presenting raw counts, the figure illustrates the typical signal magnitude of a single-shot measurement.

To clarify the independent nature of these measurements and the use of raw counts, the caption of Fig. 5 has been modified.

5. In Fig. 6(b), single- and multi-photon events are classified based on Fig. 6(a). In Fig. 6(c), the two-color separation results are presented. However, some pixel information that appears in (b) is missing in (c). This may be related to the merging of spill-over effects,

and requires further classification. In addition, the example in panels (8,10) raises a question: in (a) and (b), five connected pixels appear to form an overlapped "fuL" pattern, but they are assigned as a two-photon event. How was this assignment determined at the pixel level?

We thank the Reviewer for raising this question regarding the assignment of multi-photon events. Following this comment, we realized that the original Fig. 6(c) was inadvertently generated using a stricter certainty threshold (65%) than the rest of the manuscript (50%). We have corrected this in the revised manuscript so that the threshold is strictly consistent (50%) throughout.

However, the described phenomenon of a reduced number of illuminated pixels after color separation persists and is a direct consequence of our cluster-collapsing algorithm. When a cluster of overlapping or spread-out photon signals is reconstructed, the algorithm determines the total number and color composition of the underlying photons. This entire cluster is then collapsed into a single pixel located closest to the cluster's center of mass.

For single-photon events, this is the most straightforward approach, though theoretically, one could artificially increase the data's resolution to account for the sub-pixel information provided by the photon's spread.

To address the Reviewer's specific example of the five connected pixels at (8, 10): This assignment is not determined at the individual pixel level, but rather at the cluster level. The algorithm first identifies these five pixels as a single connected cluster. It then sums the total ADU values across all five pixels. Because this total sum corresponds to the energy of two photons, the entire five-pixel cluster is classified as a two-photon event. Finally, following our cluster-collapsing rule, this event is assigned to a single pixel at the cluster's center of mass (visualized in Fig. 6(c) with a darker red color to indicate the multi-photon count).

We opted for this center-of-mass approach to maintain algorithmic simplicity, rather than attempting to probabilistically distribute photons back to their original impact pixels. This choice is physically justified by two factors:

First, the detector's finite energy resolution inherently limits our algorithm to relatively small photon clusters. For large clusters with high photon counts, the overlapping ADU distributions make unambiguous color assignment impossible, and these ambiguous cases are discarded. Consequently, the clusters we do successfully assign are small, which inherently bounds the spatial shift introduced by collapsing them to their center of mass.

Second, this minimal spatial shift is negligible compared to the overall spatial resolution limits of the diffraction data. A non-systematic displacement of 1–2 pixels in the detector plane does not measurably alter the scattering angles or the structural features extracted during the subsequent CDI reconstruction.

To quantify this, we analyzed 10 random diffraction patterns, recording the maximum Euclidean distance between the newly assigned pixel position and the outer edge of its original cluster. As shown in the accompanying plot below, the vast majority of assigned pixels are shifted by fewer than two pixels from the cluster edge.

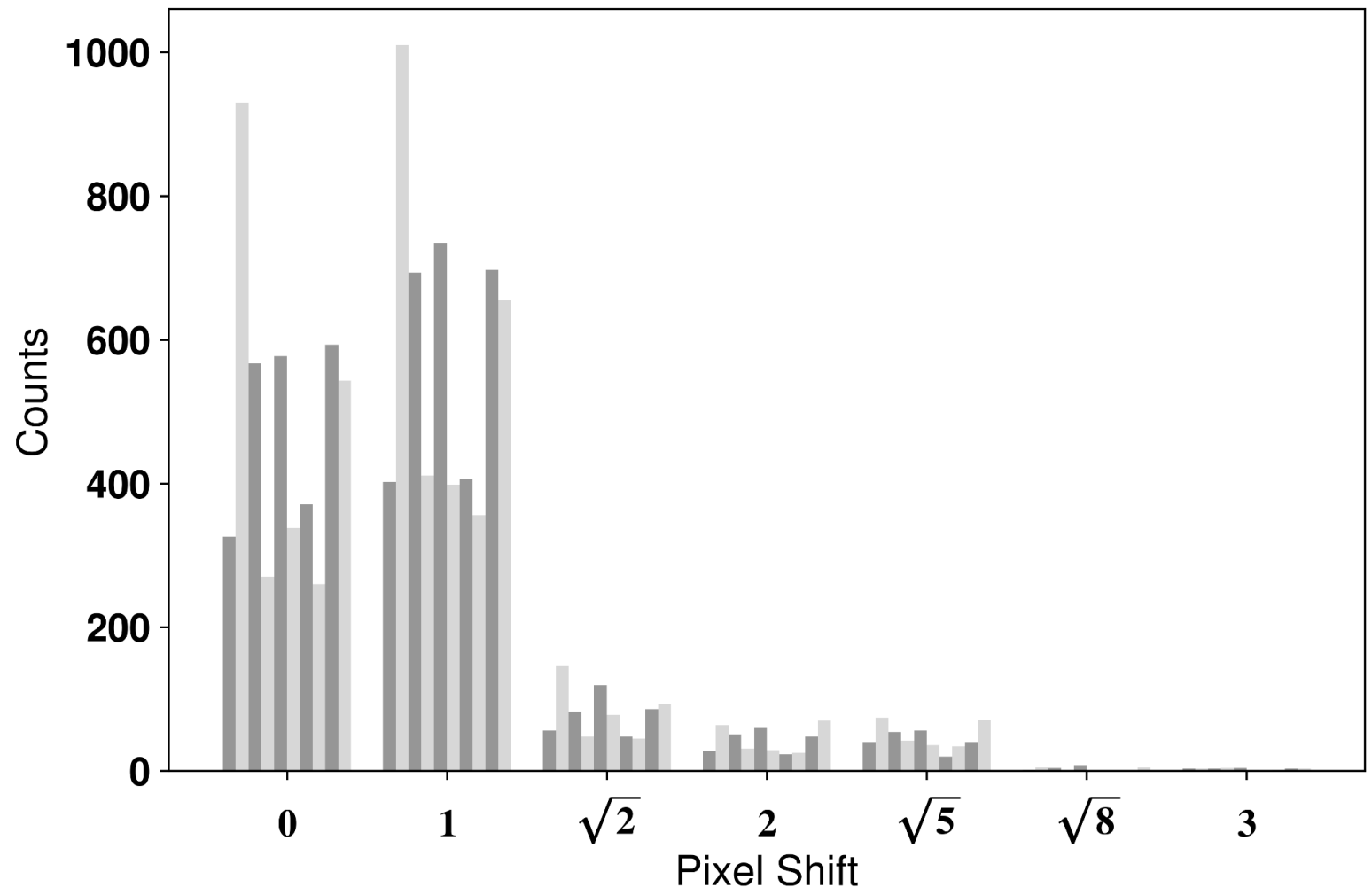

While future developments of this method might incorporate statistical assumptions to separate overlapping photons within a single cluster, it is not guaranteed that this would yield higher accuracy in every case. For instance, if a red and a blue photon land one pixel apart but spill over into the space between them, an incorrect statistical assignment of their respective colors to the constituent pixels would result in a two-pixel shift for both photons. In contrast, collapsing the entire pattern to its center of mass safely bounds the maximum positional error to one pixel for both. Therefore, we believe the choice between statistical separation and a full cluster collapse must be evaluated on a case-by-case basis depending on the specific application.

To clarify these points, we have added a detailed explanation of this aspect to the Methods section (in the paragraph on "Pixel-based Separation of Images") and to the Supplemental Material.

Furthermore, in the main text of the revised manuscript, we now state:

“Clearly, the number of illuminated pixels is significantly reduced in the color-separated images. First, ADU values are discarded if they are classified as noise or if their assignment to integer multiples of the red or blue elementary ADU values is ambiguous. Second, clusters of illuminated pixels are collapsed into the pixel position closest to the cluster's center of mass (see Methods for details).”

> *6. Regarding the probabilities, the ADU distributions for red, blue, and mixed-color cases are shown in Fig. 7. For the single-photon case, red and blue photons appear to be well separated. In the multi-photon case, however, the probability of mixed-color events is treated as a simple linear relation. In probability theory, the likelihood of obtaining a mixed-color event should generally exceed that of purely red–red or blue–blue events, assuming independent events. When deriving multi-photon probabilities from single-photon probabilities, the binomial coefficient should be included. Therefore, the analyses may need to be repeated.*

We appreciate the Reviewer’s insightful comment regarding the statistical treatment of multi-photon probabilities. We expanded the relevant section in the methods. To clarify how

these probabilities are calculated for the case of diffraction patterns, we have added a mathematical treatment in the SM and a new Supplementary Figure (Fig. SM1) showing the photon distributions for different simulated scenarios.

We agree with the Reviewer that, for a homogeneously distributed signal, the binomial coefficient must be included, thereby favoring mixed-color events. We demonstrate this statistical behavior in our new simulation of flat-field illumination and the corresponding two-photon statistics in Fig. SM.1 (a). As expected, in the case of equal probabilities for both colors, the mixed-color events have twice the probability of a double single-color event.

In our experiment, however, the signal is highly structured. The intensity ratio of the two colors varies continuously along the radial distance due to the different spatial frequencies of their respective diffraction patterns. In specific regions, such as the intensity beating zone (green-shaded area in Fig. 1), the statistical expectations are actually reversed: because the intensity maximum of one color spatially overlaps with the minimum of the other, finding a red photon in a pixel means there is a very high probability that a second photon in that same pixel will also be red and thus of the same color. This leads to a different average probability distribution, as shown in Fig. SM1(b).

In summary, because our pixel-based method is agnostic to the scatterer, we conclude that the linear combination serves as a simple and appropriate model for our experiment, and we have retained our current approach for the analysis.

> *7. In Fig. 9(b), the photon energy was scaled by a factor of 0.05 to match the scattering angle range to the experimental data due to computational limitations in MD simulations. This adjustment seems somewhat artificial and warrants further justification.*

The Reviewer correctly points out that the choice of rescaling the wavelength to calculate a diffraction pattern from the simulation is to some degree artificial. The computationally expensive simulation method does not allow us to simulate the droplet sizes present in the experiment, and the question of how to upscale the simulation results is not an easy one.

On the one hand, the dynamics in a factor 50 larger droplet will result in a larger thickness of a smoothened surface than for the simulated ~8 nm droplet. While the photon density and thus the excitation density or energy per volume in the system are the same for both the simulated and experimental droplet sizes due to the low absorption coefficient of helium, a larger particle nevertheless absorbs more photons in total and thus accumulates more charge and energy.

On the other hand, the ratio between particle size and thickness of the surface smoothening will not stay constant, either. Therefore, a direct downscaling of the wavelength to reach the size regime of the experiment, as we have done in the first version of our manuscript, will rather exaggerate the expected dynamics. In this regard, the simulations can only provide an upper limit for the expected X-ray-induced structural changes. In view of exploring regimes where structural dynamics might become observable, we even carried out the simulation at a considerably higher fluency than in the experiment.

In summary, we can only claim from the simulation of a pristine helium nanodroplet that the structural changes are expected to lie in the single-digit nanometer range and thus remain undetected within the resolution of our experiment (20 nm). Again, for the demonstration of the presented separation methods, this is an advantage because it allows us to apply the simple two-color Mie fitting routine, which is restricted to an unperturbed sphere.

In view of the Reviewer's comment, we reconsidered presenting scaled diffraction calculations of the simulated droplet in our manuscript. Instead, we carried out additional simulations for a helium nanodroplet with an identical outer radius and an embedded xenon core, now at a fluency of X-ray photons comparable to the experimental conditions. The additional simulations indicate that the expected dynamics rapidly speed up for scenarios containing highly absorbent species. While in this new scenario, the droplet was irradiated with a factor of 100 fewer photons, the thickness of the smoothened surface in the doped-droplet simulation is larger by about a factor of four. From the comparison of the two simulations, we now (more carefully) conclude that, under the current experimental conditions, pristine helium nanodroplets behave as static targets, but observing dynamics in the scientifically interesting scenario of an embedded heavy-species structure (xenon core) may be just within reach.

We updated the description in the Methods section (paragraph on "Molecular Dynamics Simulations") accordingly, now clearly stating the limitations, applicability, and reach of the simulated dynamics.

As information to the Reviewers, we note that during the same experimental campaign in which we recorded the two-color diffraction patterns of pristine helium nanodroplets discussed in the present work, we also recorded a very small data set on xenon-doped helium droplets, before a major breakdown of the FEL facility brought our experiment to an early end. The analysis of that data set, together with a complementary approach for separating the information from the two snapshots of the same particle, is presented in another work, also currently under review (Hecht, Al Haddad et al., 2026, see https://arxiv.org/abs/2508.20153).

> *8. The Supplementary Figures (Fig. SMs) are not in proper order. For example, the first supplementary figure referred at line 136 is Fig. SM7. Additionally, a caption of Fig. SM8, referenced at line 376, refers to Fig. 7, although only Fig. 3 has been introduced at that point.*

We thank the Reviewer for catching this issue. We have rearranged the Supplementary Figures accordingly to match their order of appearance in the main text.

However, the caption of the former Fig. SM8 (now Fig. SM6) still includes a forward link. To address this, we included a brief comment in the caption of Fig. SM6 that guides the reader to the Methods section. This is necessary due to the manuscript's structure in the Nature Communications format: while we present the evaluation and outlook in the main text, the details of the photon-energy-dependent separability are explained only later in the methods section.

Reviewer #3 (Remarks to the Author):

> *I co-reviewed this manuscript with one of the reviewers who provided the listed reports. This is part of the Nature Communications initiative to facilitate training in peer review and to provide appropriate recognition for Early Career Researchers who co-review manuscripts.*

We also thank the third Reviewer for co-reviewing the manuscript and for their valuable suggestions and advice.

Response to reviews on manuscript NCOMMS-25-78444A.

We thank the Reviewers for their further evaluation of our manuscript. We are pleased that the previous revisions have addressed the majority of the concerns.

Reviewer #1 (Remarks to the Author):

> *All my previous comments have been addressed satisfactorily, and the paper can be accepted from my perspective.*

We thank the Reviewer for their positive assessment of our revised manuscript and for recommending its publication.

Reviewer #2 (Remarks to the Author):

> *I thank the authors for their careful and detailed responses to the original comments. Overall, the manuscript has been substantially improved, and most of my concerns have been satisfactorily addressed. Two minor points remain on #5, which I would ask the authors to clarify before publication.*
> *The authors' explanation of the cluster-collapsing algorithm — in which a connected cluster is identified, its total ADU value is summed, the number and color composition of photons is determined at the cluster level, and the result is collapsed onto the pixel nearest the cluster's center of mass — clearly answers how the two-photon event at (8,10) is assigned. The correction of the certainty-threshold inconsistency (from 65% back to 50%) is also appropriate. On this primary point, the response is satisfactory.*
>
> *Two concerns remain, however, that I would like the authors to address:*
> *(a) Potential internal tension between the arguments in Comments 2 and 5. In their response to #2, the authors argue that clean wavelength separation at high-q will strongly benefit imaging and will help determine accurate structural features within a multi-wavelength phase-retrieval framework. In the present response, however, the 1–2 pixel displacement introduced by the center-of-mass assignment is justified by the statement that "a non-systematic displacement of 1–2 pixels in the detector plane does not measurably alter the scattering angles or the structural features extracted during the subsequent CDI reconstruction." These two positions appear to be in tension: if high-q separation is critical for resolution, a 1–2 pixel positional error in exactly that region cannot be dismissed as negligible. I ask the authors to reconcile these two statements explicitly in the revised text — for instance, by quantifying the regime in which the 1–2 pixel shift is indeed negligible, and by clarifying where and how this condition is compatible with the resolution benefits claimed in Comment 2.*

We thank the Reviewer for pointing out this remaining apparent inconsistency following from two seemingly contradicting lines of argument. As we will discuss below, in our case, the two statements, (1) clean color separation in the high-q regime, possible through our pixel-based color separation method, allows for extracting high-resolution information, and (2) a non-systematic displacement of one or two pixels does not hamper the extraction of structural features during a subsequent CDI reconstruction, are indeed not contradictory. As

we will show below, in our case, the exact position of single photons at the single-pixel level is not a limiting factor for real-space resolution in this regime. We acknowledge that for certain applications, further improvement of the placement algorithm can be beneficial or even required. As discussed extensively in our previous response, a path towards such a more highly resolved placement algorithm is, however, not straightforward and will depend on making more assumptions. In consequence, our current approach already provides the best possible model-free solution to the problem.

The apparent tension between these arguments is resolved by comparing the magnitude of the positional shift to the signal's intrinsic oscillation period, the coordinate jitter induced by the FEL bandwidth, and the inherent robustness of high-q features to absolute pixel uncertainties.

An intrinsic feature of single-particle CDI is that the signal in the diffraction pattern changes on scales that have a lower bound. This is strictly related to the isolated nature of the sample, having a limited size. The Fourier transform of the single-particle diffraction pattern, also called its autocorrelation, is characterized by a maximum frequency. This maximum extension of the autocorrelation function or maximum frequency at which the diffraction signal can change on the detector as a function of the momentum transfer q, in consequence, constrains a lower limit Δq for the variation of the signal on the detector.

This maximum allowed frequency can be straightforwardly quantified as $\frac{2\pi}{D_{max}/2}$, where D_{max} is the maximum extension of the autocorrelation function. For a spherical droplet, D_{max} corresponds to twice the particle diameter, i.e., $D_{max} = 4R$. The fastest oscillation in the signal thus happens on a reciprocal length $\Delta q = \frac{\pi}{R}$. This length can be translated to number of pixels by calculating the size in reciprocal space of a single pixel $\delta q = k\,\delta\theta = \frac{2\pi}{\lambda}\delta\theta$. The fastest signal oscillation in terms of number of pixels, M, on the detector for a given particle with radius R is thus $M(R) = \frac{\Delta q}{\delta q} = \frac{\lambda}{2R\,\delta\theta}$.

In the geometry of this experiment $\delta\theta = 2.18 \cdot 10^{4}$. At 1 keV photon energy ($\lambda = 1.24$), the fastest signal oscillation thus covers $M(R = 354\,nm) = 8.0$ pixel for the average size of the droplets in our manuscript and still covers $M(R = 850\,nm) = 3.3$ pixel for the biggest recorded particles.

The introduction of a non-systematic uncertainty of 1-2 pixel in the position assignment for the two-photon events can be modeled as a point spread function (PSF) in the recorded data with $\sigma = 1$ pixel. This “smoothing” of the diffraction signal is much smaller than the calculated minimum oscillation period for the average droplet size (354 nm) of 8 pixel, and still significantly smaller than 3.3 pixel calculated for the largest droplet recorded. This means that even the 1-2 pixel uncertainty fully preserves the signal properties without significant loss of information.

On top of that, this increased uncertainty affects only the two-photon events, which represent only ~10% of the pixel-wise separated signal. Single-photon events, which represent the great majority of the color-separated information, are always assigned to a specific pixel

coordinate. This means that they have a maximum uncertainty corresponding to $\frac{1}{\sqrt{2}}$, i.e., half the diagonal of a single pixel. The corresponding PSF is thus characterized by $\sigma \ll 1$, with no influence on signal fluctuations that take place over several pixels.
To summarize, the uncertainty of the photon placement in the detector coordinates is significantly smaller than the minimum oscillation length-scales of the recorded signal, and does not alter the contained information.

A second aspect of the referee question concerns the effects of an uncertainty at high-q, where intuition suggests that even a small uncertainty would introduce large errors. As discussed above, an isolated particle has a smallest signal oscillation. This lower bound is valid throughout the whole pattern at all q values. This is the reason why the spacing of the diffraction rings produced by a droplet stays constant on the detector, and it is independent of the magnitude of q.

However, there are cases where the sample is not isolated, and this assumption does not hold. A straightforward example is the application of the color-separation method to crystallography, where the position of a Bragg peak q_p is related to a specific lattice size x_p via the relationship $x_p = \frac{2\pi}{q_p}$. Via error propagation, it is straightforward to calculate that an uncertainty Δq carries an uncertainty in x_p equal to $\Delta x = \frac{2\pi}{q_p^2}\Delta q$. An uncertainty in the photon's placement corresponds to a fixed value of Δq independent of the position in the detector, while the propagated error on the size estimate Δx is reduced by a factor q_p^2 .
To put it into perspective, a one-pixel error at a low scattering angle, e.g., 0.3 deg, corresponds to a 10 nm uncertainty in x_p. Conversely, the same misplacement of the photon at 3° scattering angle brings only a 1Å uncertainty.

To summarize, the calculations shown above lead to the counterintuitive conclusion that a misplacement of a photon event in the high-q region has a much smaller impact than the same misplacement in low-q regions, i.e., the high-resolution region of the detector is much less sensitive to the exact photon position.

Finally, the intrinsic 1% bandwidth of the FEL already introduces a coordinate jitter of ~1 pixel at this experiment’s high scattering angles, making the assignment shift commensurate with existing physical limits.

To explicitly reconcile these points for the reader, we have added a summary of the above in the Supplemental Material and the following clarifying paragraph to the manuscript:

“The resulting average shift of below one pixel remains well below the information limit defined by the diffraction fringe width and the intrinsic coordinate jitter from the FEL bandwidth. Furthermore, the sensitivity of real-space features to absolute pixel errors scales as q^{-2}, ensuring that the high-resolution information at high-q is inherently robust to minor positional uncertainties. We note that while this shift is negligible for oversampled, isolated-sample CDI, its impact would be more significant in experiments sensitive to

absolute peak positions, such as Bragg diffraction or crystallography (see SM for a detailed quantification).”

> *(b) Inconsistency between the response letter and the revised manuscript. The authors quote the following sentence as having been inserted into the main text:*
> *"Clearly, the number of illuminated pixels is significantly reduced in the color-separated images. First, ADU values are discarded if they are classified as noise or if their assignment to integer multiples of the red or blue elementary ADU values is ambiguous. Second, clusters of illuminated pixels are collapsed into the pixel position closest to the cluster's center of mass (see Methods for details)."*
> *In the revised manuscript (around line 256), the inserted wording conveys a similar meaning but does not precisely match the text given in the response letter. Please verify that the response letter and the final manuscript are fully consistent.*

We thank the reviewer for noting the discrepancy. It was an oversight in the previous response letter. During the multi-author revision process for Revision 1, the manuscript text was iteratively refined to ensure consistency and more common terminology, e.g., “hit pixels”. However, an older version of that sentence had inadvertently remained in our response letter.

The text in the manuscript remains unchanged between Revision 1 and Revision 2. We have now verified that the current response letter and the manuscript are fully consistent. The wording in the manuscript is as follows:

“Clearly, the number of assigned pixels is significantly reduced in the color-separated images. First, signal is discarded if classified as noise or if the assignment to integer multiples of the red or blue elementary ADU values is ambiguous. Second, clusters of hit pixels are collapsed into the pixel position closest to the cluster's center of mass (see Methods for details).”

We believe these clarifications and the additional analysis in the Supplemental Material fully address the Reviewer's concerns and demonstrate the reliability of our color-separation approach. We thank the Reviewer again for their constructive feedback, which has significantly strengthened the manuscript.
